# SEM mapping of sequence-specific protein–DNA interactions on long DNA molecules

**DOI:** 10.1093/nar/gkag687

**Published:** 2026-07-09

**Authors:** Chanyoung Noh, Sangwon Lee, Yoonjung Kang, Taesoo Kim, Taebin Yun, Yoojin Kim, Gyuri Park, Priyannth R Sundharbaabu, Sang-Hee Shim, Kwang-il Lim, Jung Heon Lee, Kyubong Jo

**Affiliations:** Department of Chemistry, Sogang University, Seoul 04107, Korea; Department of Chemistry, Sogang University, Seoul 04107, Korea; Department of Chemistry, Sogang University, Seoul 04107, Korea; Department of Chemistry, Sogang University, Seoul 04107, Korea; Department of Chemistry, Sogang University, Seoul 04107, Korea; Department of Chemistry, Sogang University, Seoul 04107, Korea; Department of Chemistry, Sogang University, Seoul 04107, Korea; School of Advanced Materials Science and Engineering, Sungkyunkwan University (SKKU), Suwon 16419, Korea; Department of Chemistry, Korea University, Seoul 02841, Korea; Department of Chemical and Biological Engineering, Sookmyung Women’s University, Seoul 04312, Korea; School of Advanced Materials Science and Engineering, Sungkyunkwan University (SKKU), Suwon 16419, Korea; Department of MetaBioHealth, Sungkyunkwan University, Suwon 16419, Korea; Department of Chemistry, Sogang University, Seoul 04107, Korea; Center for Nano Materials, Sogang University, Seoul 04107, Korea

## Abstract

Direct visualization of protein-binding positions along individual DNA molecules provides direct readouts of binding location, occupancy, and heterogeneity beyond the reach of ensemble assays. However, existing single-molecule imaging methods face a persistent trade-off between spatial resolution, field of view, and throughput. Here, we establish an scanning electron microscopy (SEM)-based approach that combines contrast enhancement with sequence-defined labeling to image extended DNA molecules and resolve protein-bound regions along their contour. We validate this capability across distinct binding regimes, including sequence-defined streptavidin–fluorescent protein labels on biotinylated λ DNA, mapping of densely bound dCas9 regions on plasmid DNA, and machine-learning–assisted detection of localized dCas9 binding on human genomic DNA (F1 = 0.97). SEM achieves mean positional offsets of 410 ± 325 bp for nick-translated labels and 116 ± 63 bp for dCas9-bound regions, ~3-fold improved over fluorescence imaging, while supporting large-area surveys of extended DNA molecules across multi-scale magnifications—a capability not accessible by transmission electron microscopy or atomic force microscopy. These results establish SEM as a scalable platform for simultaneous structural visualization and quantitative mapping of sequence-specific DNA–protein interactions along individual DNA molecules.

## Graphical Abstract

**Graphical Abstract:**
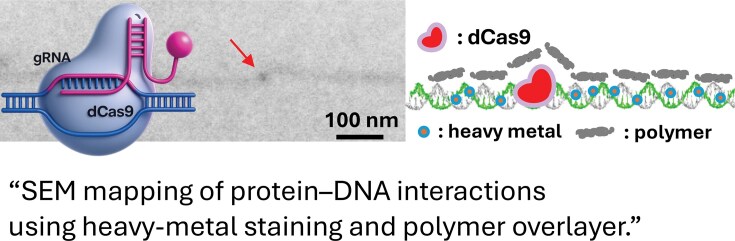

## Introduction

Visualization of DNA at the single-molecule level enables direct assessment of structural and conformational features—such as contour length, bending flexibility, and local compaction—that are critical for understanding genome organization and function. Beyond static structure, this approach allows direct observation of molecular processes involving DNA, including sequence-specific protein binding, enzymatic modification, and recombination. By capturing these interactions along individual DNA strands, single-molecule imaging reveals mechanistic details of DNA recognition and processing that are obscured in bulk assays. Over the past decades, a variety of imaging methods, including fluorescence microscopy [1], atomic force microscopy (AFM) [2], and electron microscopy [3], have been employed to study DNA molecules. Among these, fluorescence microscopy has become a widely used platform for versatile and selective visualization of DNA and associated molecular processes, with ready compatibility with microfluidic platforms [4–8]. However, its spatial resolution is fundamentally constrained by optical diffraction. Super-resolution microscopy partially overcomes this limitation by improving localization precision to the nanometer scale [9–11], yet typically requires complex optical setups, offers limited fields of view, and suffers from photobleaching and long acquisition times. AFM provides nanometer-resolution topographic imaging of DNA and numerous protein–DNA complexes [2, 12–14], although its probe-based raster scanning limits imaging speed and, in many cases, makes large-area searches for sparse molecules challenging. High-speed AFM (HS-AFM) has enabled direct visualization of dynamic processes such as Cas9-mediated DNA cleavage with exceptional spatial and temporal resolution [15], but the technical complexity of these measurements currently restricts their use to specialized systems.

Transmission electron microscopy (TEM) has a long history in DNA visualization [3, 16, 17] and can achieve spatial resolutions approaching 20 pm under optimized conditions [18]. Because DNA is composed of light elements with weak electron scattering, contrast is typically enhanced using heavy-metal stains such as uranyl acetate [19]. Regulatory restrictions on uranyl compounds have motivated the development of alternative reagents, including UranyLess [20], which employs lanthanide cations to generate comparable contrast. Conventional TEM relies on thin carbon support films to permit electron transmission. While effective for imaging, these films provide limited control over DNA adsorption, exhibit substantial heterogeneity, and are poorly compatible with surface functionalization or integration into microfluidic platforms. As a result, TEM-based approaches are inherently constrained for scalable and functional single-molecule DNA analysis, motivating complementary strategies that enable direct DNA imaging on rigid, planar substrates such as glass or silicon.

Scanning electron microscopy (SEM) provides a complementary imaging modality by detecting secondary electrons emitted from solid substrates rather than transmitted electrons [21]. Planar substrates such as silicon wafers are smooth, chemically versatile, and readily functionalized using established silane chemistries, enabling molecular patterning and microfluidic integration. Owing to the presence of a native silicon oxide layer, silicon surfaces share many advantageous properties with glass while offering improved electrical conductivity. This oxide layer supports silane-based functionalization, while the conductive nature of silicon mitigates charging effects during SEM imaging, enabling stable, high-contrast visualization [22]. Modern field-emission SEM instruments routinely achieve sub-nanometer resolution (typically < 1 nm) [23], well below the diameter of a DNA duplex (∼2 nm). In addition, SEM enables imaging across a magnification range spanning more than four orders of magnitude (typically × 10 to > × 100 000), allowing both large-area surveys for sparse molecule identification and high-resolution imaging of individual targets through automated stage scanning.

However, a key question remains: how can DNA molecules be visualized under SEM? Because DNA is composed of low-atomic-number elements, it produces weak intrinsic secondary-electron contrast. One strategy is to attach electron-dense labels to DNA, as demonstrated in nanoparticle-based labeling approaches [24–26]. Another strategy is to attenuate substrate-derived signal beneath the DNA. We previously achieved this using DNA–protein–polymer assemblies in which protein components bridge DNA to polyvinylpyrrolidone (PVP), yielding dark-line contrast on silicon [27, 28]. Building on this principle, we asked whether heavy-metal stains commonly used in TEM could generate similar contrast in SEM by either enhancing or suppressing secondary electron emission, which would cause DNA to appear bright or dark in SEM images. Either mode of contrast would provide informative readouts for single-molecule DNA visualization.

Here, we establish an SEM-based imaging framework that enables direct visualization of DNA structure and associated proteins on individual molecules. Building on our previously developed metal-free protein–polymer staining [27, 28], we incorporate the heavy-metal reagent UranyLess, a proprietary lanthanide mix containing La^3+^ and Gd^3+^ [29], to substantially enhance DNA contrast on silicon substrates and to reveal protein-bound regions as locally modulated SEM features. Using this framework, we first visualize sequence-defined protein-labeled tracts generated by nick translation that support contour-based positional mapping, and then extend the same strategy to direct sequence-specific protein–DNA interactions, from densely bound dCas9 regions on plasmid DNA to individual sparse dCas9 binding events resolved as discrete features along the DNA contour on human genomic DNA. Quantitative comparison with fluorescence microscopy demonstrates three-fold improvements in positional accuracy and precision, with machine-learning–assisted detection of localized dCas9-associated features achieving F1 = 0.97, defined as the harmonic mean of precision and recall. These demonstrations establish an SEM-based platform for practical, high-resolution single-molecule imaging and base-pair-resolved positional mapping of sequence-specific protein–DNA interactions.

## Materials and methods

### DNA samples and proteins

Bacteriophage λ DNA (48.5 kb), pWY82 plasmid DNA (11.6 kb), and human genomic DNA (HG002) were used as templates. Large genomic DNA from human cells was isolated using agarose gel plugs, followed by proteinase K digestion and β-agarase I recovery. Fluorescent protein–DNA-binding proteins (tTALE–mNeonGreen) [30] and streptavidin–fluorescent proteins (SA-RRvT and SA-eGFP) [31] were expressed in *Escherichia coli* BL21(DE3) and purified by Ni–NTA affinity chromatography. dCas9–gRNA ribonucleoproteins were assembled from SNAP-tagged dCas9 (NEB) and annealed crRNA:tracrRNA duplexes (IDT) [32]. SA-FP–labeled DNA was generated by nick translation with Nb.BssSI and DNA polymerase I on λ DNA, followed by biotin-dUTP incorporation and subsequent SA-FP conjugation [31].

### Surface preparation and microfluidics

Positively charged silicon wafers were generated by silanization with N-trimethoxysilylpropyl-N,N,N-trimethylammonium chloride [33]. DNA samples (1 μl) were loaded into polydimethylsiloxane (PDMS) microchannels (100 μm × 2.4 μm) to align the DNA molecules, followed by air drying for 50 min before removal of the PDMS device [34].

### SEM imaging

DNA was deposited on functionalized silicon wafers, and SEM images were acquired using an Apreo 2S HiVac microscope (Thermo Fisher) operated at accelerating voltages of 10–30 kV with a secondary electron detector. Samples were imaged under high-vacuum conditions without conductive metal coating. Three DNA staining conditions were applied: (i) UranyLess; (ii) UranyLess and PVP; and (iii) UranyLess and DNA-binding protein–PVP.

### Image analysis

Building on automated DNA backbone tracing methods developed for AFM [35, 36], we traced contours in SEM and TEM images using a dynamic-programming-based optimal-path algorithm [37, 38] that maximizes line-strength intensity, applied to CLAHE-enhanced [39] and Meijering-filtered [40] line-strength maps. The trace with the higher mean dark-path intensity was selected automatically. At each trace point, a 41-pixel intensity profile was extracted perpendicular to the local tangent. For direct comparison across magnifications, per-point profiles were resampled onto a common nm grid (±65 nm, 41 samples). Apparent DNA edge-to-edge width was measured on the per-run averaged profile by sub-pixel localization of the intensity-gradient extrema.

For machine-learning-based detection of protein-associated signals, each profile was summarized by 82 scale-invariant features derived from the edge-baseline-normalized trace, including multi-scale central-window intensity descriptors, first- and second-derivative features, peak-relative widths, left/right asymmetry, and statistical moments. A calibrated random-forest [41] classifier with sigmoid calibration [42, 43] implemented in scikit-learn [44] was trained on 13 147 profiles (3551 positive and 9596 negative) from 13 manually annotated images, using 600 trees, balanced-subsample class weighting, and five-fold internal cross-validation. Leave-one-positive-image-out cross-validation was used to prevent data leakage and to evaluate out-of-fold probabilities. The along-trace Gaussian smoothing window, minimum run-length filter, and decision threshold were jointly grid-searched on the cross-validation probabilities to maximize F1 score, yielding a final setting of ~38 nm smoothing, 24 nm minimum run length, and a decision threshold of 0.35. Out-of-fold performance was F1 = 0.97, with precision = 0.97 and recall = 0.98.

Protein-binding signals were further compared between modalities by their center-localization precision and apparent footprint. The localization precision along the DNA contour (σloc) of each binding site was estimated as the standard error of the fitted Gaussian center derived from the fit covariance, providing a covariance-based estimate of localization uncertainty [45, 46]. Because this estimate depends on both the fitted signal width and local noise level, σloc reflects the combined effect of signal width and signal-to-noise ratio [47]. The apparent width was defined as the FWHM of the center-registered, averaged protein-associated feature profile. For fluorescence microscopy, only spatially isolated foci were analyzed (*n* = 50 from three single-gRNA images). For SEM, protein-associated features were analyzed from 32 segments across 10 SEM images after subtracting the DNA-backbone signal from each cross-section to isolate the protein-associated contribution. Full implementation and all analysis code are available on GitHub. https://github.com/jolaboffice/SEM_DNA_Analysis

Further experimental details are provided in the Supplementary Information Methods.

## Results

### From TEM to SEM: DNA visualization with heavy-metal staining

**Figure 1.**
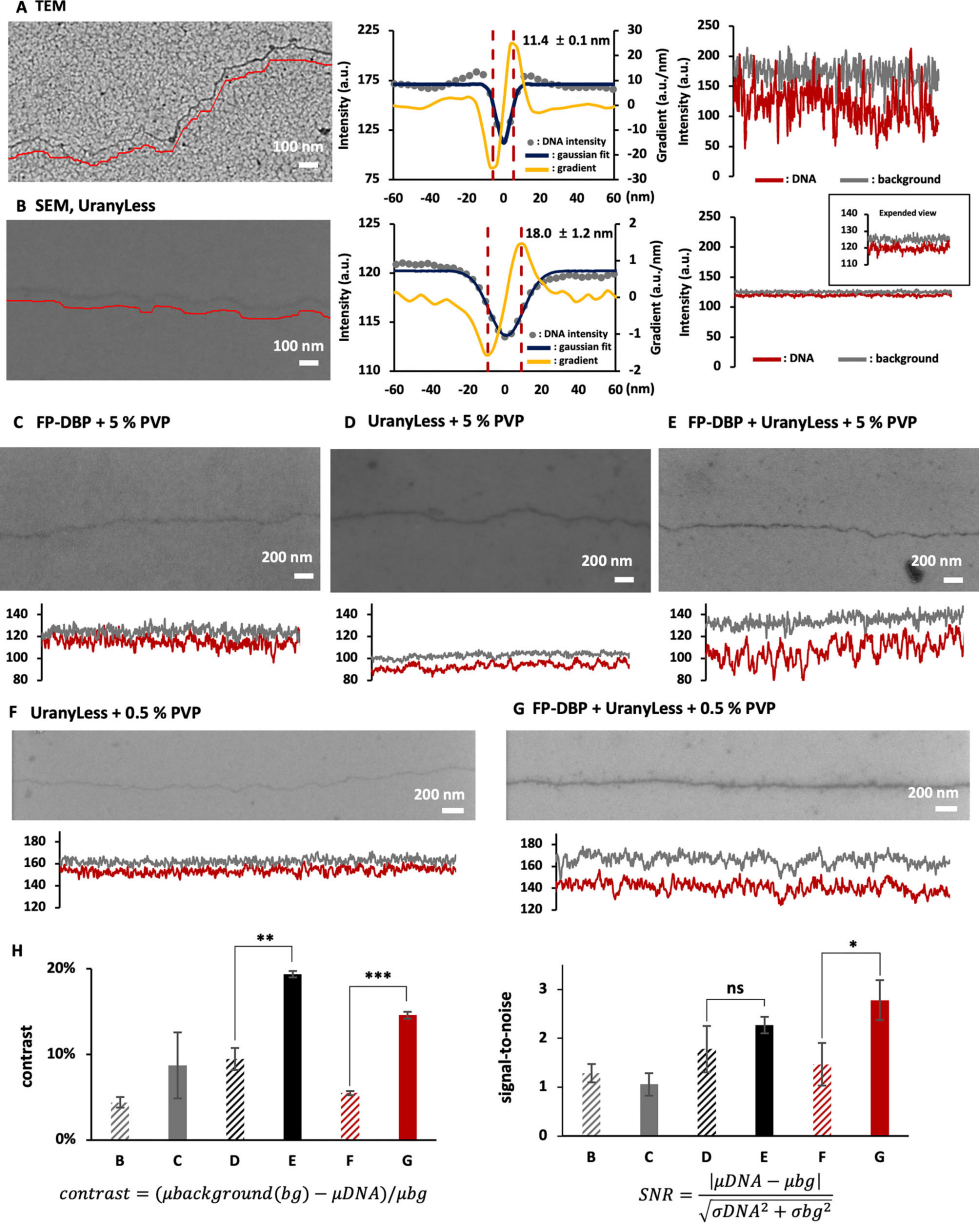
Quantitative DNA imaging in TEM and SEM under UranyLess-, PVP-, and FP-DBP-based conditions. UranyLess-stained DNA imaged by TEM on carbon film (**A**) and SEM on silicon wafer (**B**). DNA backbone contour is indicated by an offset trace. Transverse intensity profiles with gradient analysis yielded apparent widths of 11.4 ± 0.1 nm (A, *n* = 500) and 18.0 ± 1.2 nm (B, *n* = 1000). Longitudinal DNA and background traces shown on the right; expanded inset in panel (B). SEM images and corresponding longitudinal intensity traces of DNA acquired under different coating conditions: (**C**) FP-DBP (tTALE–mNeonGreen) + 5% PVP, (**D**) UranyLess + 5% PVP, (**E**) FP-DBP + UranyLess + 5% PVP, (**F**) UranyLess + 0.5% PVP, and (**G**) FP-DBP + UranyLess + 0.5% PVP. The inset in panel (F) shows an expanded view of the DNA and background intensity traces. (**H**) Weber contrast and signal-to-noise ratio (SNR) for the SEM conditions shown in panels (B–G). Data are presented as mean (*µ*) ± SD from three independent experiments (*n* = 3). Statistical comparisons were performed using Welch’s *t*-test. **P < .01, ***P < .001, *P < .05, ns: not significant.

A TEM image of a DNA molecule stained with a heavy-metal reagent (UranyLess) is shown in Fig. 1A. Consistent with conventional TEM contrast mechanisms, the DNA backbone appears as a dark line due to strong electron attenuation by bound heavy-metal ions. We therefore tested whether the heavy-metal ions in UranyLess could generate contrast in SEM by either enhancing secondary electron emission from the DNA or suppressing emission from the underlying silicon substrate. In the former case, the DNA backbone would appear as a bright line, whereas in the latter case it would appear as a dark line. To distinguish between these two mechanisms, we performed SEM imaging of UranyLess-stained DNA immobilized on a silicon wafer. The resulting image (Fig. 1B) showed DNA as a dark line, indicating that secondary electron emission from the silicon beneath the DNA was suppressed. However, the contrast was lower than that observed in Fig. 1C, which was obtained using a combination of DNA-binding protein (FP-DBP) and PVP as reported previously [27, 28], in which PVP forms an organic overlayer that blocks secondary electrons emitted from the semiconductive silicon surface, while FP-DBPs mediate physical association between DNA and PVP, as PVP itself lacks intrinsic DNA-binding affinity. Based on these observations, we tested whether combining UranyLess with PVP and FP-DBP could further enhance DNA visibility. When DNA was treated with UranyLess and PVP, strands exhibited substantially improved contrast (Fig. 1D) even without DNA-binding proteins, suggesting that PVP can associate noncovalently with UranyLess-coated DNA. The full combination of UranyLess, PVP, and FP-DBPs provided the strongest and most stable contrast (Fig. 1E), reflecting enhanced secondary electron suppression by the heavy metal–polymer layer together with protein-mediated stabilization of the DNA on the surface. To further examine the origin of UranyLess-based contrast, we analyzed UranyLess on silicon by EDS and compared its performance with related lanthanide salts (Supplementary Fig. S1), supporting contributions from both lanthanide ions and associated reagent components. Detailed analysis procedures are provided in the Methods and Supplementary Figs S2 and S3, with additional measurements from three independent images summarized in Supplementary Fig. S3.

SEM provided substantially more stable and reliable signal profiles than TEM. In Fig. 1A, intensity profiles along the DNA backbone are partially mixed with background signals, reflecting pronounced background fluctuations that reduce signal detectability. In contrast, SEM imaging produced well-separated DNA and background intensity profiles. Notably, under the UranyLess + PVP condition (Fig. 1D), DNA and background intensity profiles are well separated with minimal overlap. This separation becomes even more pronounced in the presence of DNA-binding proteins (Fig. 1E), where protein-associated signals are clearly distinguishable. Under this condition, the intensity profile along the DNA backbone exhibits substantial fluctuations. These fluctuations likely arise from non-uniform protein binding along the DNA molecule, suggesting that the observed signal variations reflect underlying protein–DNA interaction patterns at the single-molecule level.

To further enhance this effect and identify coating conditions that best reveal protein-associated contrast, we tested reduced PVP concentrations in the presence or absence of FP-DBP. Among them, 0.5% PVP produced the most pronounced FP-DBP-dependent contrast enhancement. A moderate PVP concentration maximizes differential visibility between DNA and protein-bound regions, providing an optimal condition for SEM imaging of protein-associated DNA structures. This behavior is likely due to the higher affinity of PVP for the protein surface compared with bare DNA or UranyLess-coated DNA, leading to more effective polymer association at intermediate concentrations. In the absence of FP-DBP, UranyLess + 0.5% PVP yielded relatively weak DNA–background separation, comparable to UranyLess alone (Fig. 1F). Upon FP-DBP addition, however, both SEM images and corresponding longitudinal intensity profiles showed markedly improved separation, enabling clear discrimination between protein-bound and protein-free DNA molecules (Fig. 1G). Consistent with these observations, both Weber contrast and signal-to-noise analyses indicate that 0.5% PVP provides the strongest FP-DBP-dependent contrast enhancement, with statistically significant differences observed between selected conditions (Fig. 1H) [33, 48].

### SEM imaging of sequence-defined protein labels

The use of a heavy-metal staining reagent enables SEM visualization of DNA without the need for DNA-binding proteins as mediators between DNA and the coating layer, thereby allowing such proteins to function as independent labeling reagents. In the present study, the FP-DBP (tTALE–mNeonGreen [30]) binds uniformly along the DNA, producing a consistently thickened backbone and corresponding intensity fluctuations. These results indicate that protein-associated contrast can be detected along individual DNA molecules. By contrast, sequence-specific binding proteins are expected to generate localized contrast enhancements at their binding sites. We therefore extended this approach to sequence-specific protein labeling and tested whether sequence-defined protein features can be visualized as localized dark regions along DNA for SEM-based genome mapping.

**Figure 2.**
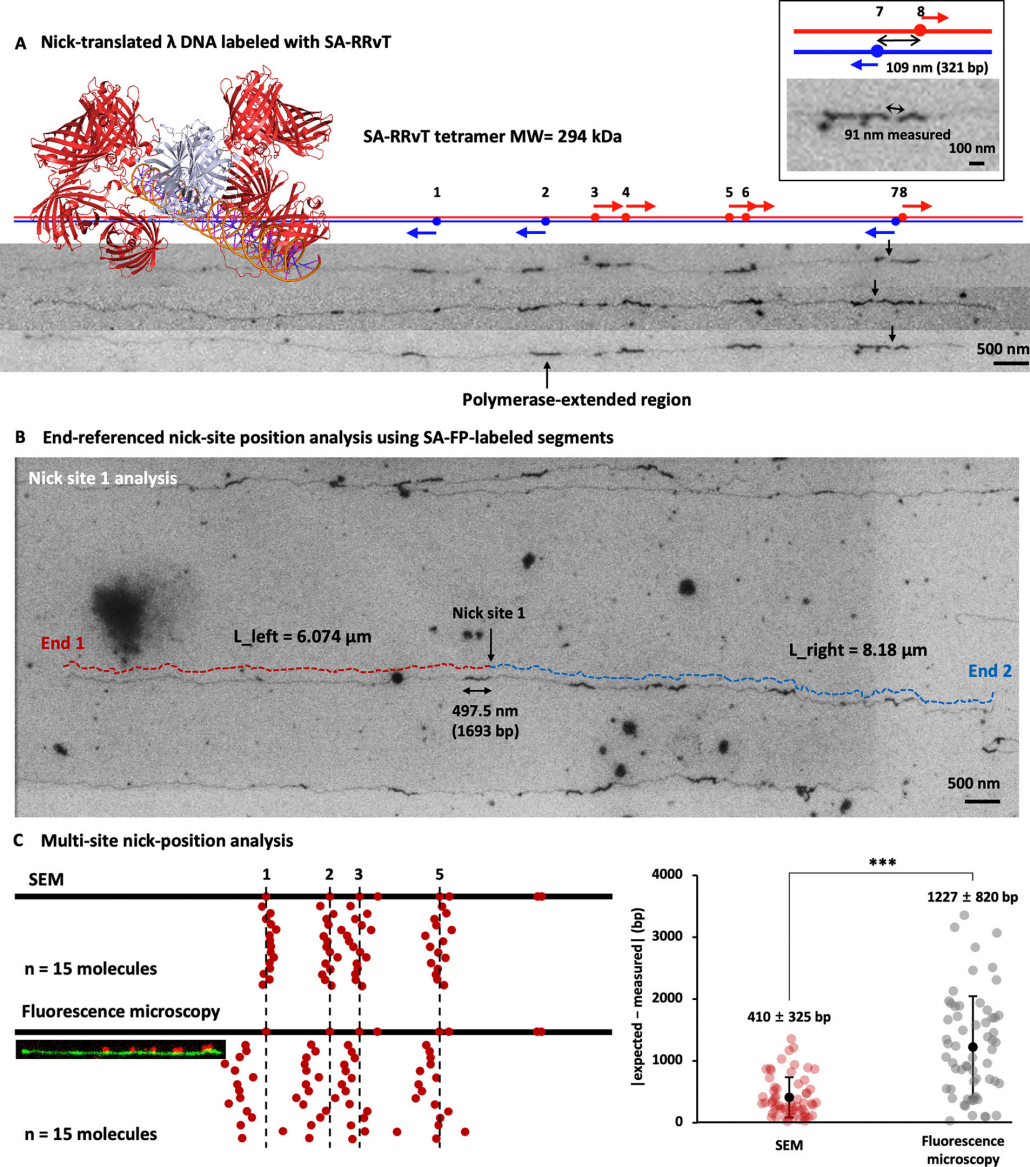
SEM mapping of nick-translated DNA labels. (**A**) SEM images of nick-translated λ DNA labeled with streptavidin (SA)–RRvT. A structural model of the SA-RRvT tetramer is shown on the left. The schematic indicates the expected labeled sites and the direction of polymerase-mediated extension from each nick site. In SEM, the labeled regions appear as short darkened tracts along the DNA backbone. The arrow indicates a representative polymerase-extended region. The inset compares two nearby labeled sites (sites 7 and 8), showing an expected spacing of 109 nm (321 bp) and a measured separation of 91 nm in SEM. Black arrows in the main SEM panel mark the resolved site 7–8 regions corresponding to the closely spaced labels shown in the inset. (**B**) End-referenced nick-site position analysis using SA-FP-labeled segments. This panel shows an analysis of nick site 1. In the upper panel, contour distances from the labeled segment to the two DNA ends were measured as L_left and L_right. Because nick translation generates an extended labeled tract rather than a point-like signal, the nick-site position was inferred from the segment boundary corresponding to the known direction of polymerase extension. In the representative molecule shown, the darkened segment associated with the indicated nick site measured 497.5 nm, corresponding to 1693 bp equivalent after contour-normalized conversion using the molecule-specific contour length. (**C**) Multi-site nick-position analysis. The same end-referenced analysis was applied to nick sites 1, 2, 3, and 5, and the measured positions were read out in base-pair-equivalent coordinates by first calculating raw fractional contour positions from the measured end-referenced contour lengths and then converting these values using the corresponding reference length. A representative fluorescence image is shown below. For SEM, nick-site positions were inferred from the boundaries of resolved SA-FP-labeled segments, whereas fluorescence positions were defined by the centroids of diffraction-limited fluorescence signals. The plot on the right compares the absolute offsets between the measured and expected positions pooled across nick sites 1, 2, 3, and 5 from 15 molecules per modality, yielding 60 site-level measurements for each modality. Black points and error bars indicate mean ± SD of absolute offsets. Welch’s *t*-test. ***P < .001.

To test this concept, we performed SEM positional mapping of nick-translated DNA labels using λ DNA (48.5 kb), adapting a nick-labeling strategy widely used in optical mapping [49, 50]. In this approach, biotin labeling was introduced by nick translation at predefined sites (C^TCGTG) on double-stranded λ DNA, followed by visualization with a streptavidin–fluorescent protein conjugate (SA-RRvT) [31]. Because SA-RRvT forms a tetrameric complex of tandem-dimeric RRvT units (overall octameric; ∼294 kDa, Fig. 2A), it serves as an ideal model for testing whether sequence-defined protein labels can be detected by SEM. The nicking endonuclease Nb.BssSI introduced single-strand nicks at its recognition motifs, serving as initiation sites for polymerase-mediated biotin-dUTP incorporation. Subsequent binding of SA-RRvT to the incorporated biotin generated discrete fluorescence signals and corresponding darkened features along the DNA backbone in SEM. Because polymerase extends biotinylated nucleotides away from the nick site, labeled sites appeared as short darkened tracts rather than point-like marks. In the representative molecule shown in Fig. 2B, the darkened segment associated with the indicated nick site measured 497.5 nm, corresponding to 1693 bp equivalent after contour-normalized conversion using the molecule-specific contour length. This extended labeling length likely explains why nearby sites 5 and 6 (expected spacing of 789 bp), whose polymerase extension proceeds in the same direction, appear as a single merged segment. Nevertheless, two nearby labeled sites whose polymerase extension proceeded in opposite directions (sites 7 and 8) remained distinguishable in SEM, with a measured spacing of 91 nm compared with an expected spacing of 109 nm (Fig. 2A, inset). Similar sequence-specific labeling behavior was also observed with SA-eGFP (Supplementary Fig. S4A). To further assess the reproducibility of this closely spaced label pair, we quantified the site 7–8 gap across SA-FP-labeled λ DNA molecules. The site 7–8 separation was resolved in 21 of 23 analyzed molecules (91%), and the measured gap was 99.9 ± 17.1 nm (mean ± SD, *n* = 21), close to the expected spacing of 109 nm (Supplementary Fig. S4B).

These SA-FP-labeled tracts also supported SEM-based genome mapping with improved positional accuracy and precision compared to fluorescence microscopy (Fig. 2B). We first established the analysis workflow using nick site 1 as an example (Fig. 2B). In this analysis, the contour distances from the labeled segment to the two DNA ends were measured on individual molecules. Because nick translation generates short labeled segments rather than point-like signals, the nick-site position was inferred from the segment boundary corresponding to the known direction of polymerase extension. Raw fractional contour positions were first calculated from the measured end-referenced contour lengths and then converted to bp-equivalent coordinates using the corresponding λ DNA reference length. This end-referenced analysis was then extended to multiple nick sites, including sites 1, 2, 3, and 5 (Fig. 2C). In SEM, positions clustered near the expected nick-site coordinates, whereas fluorescence microscopy provided centroid-based positions from diffraction-limited signals and showed larger deviations from the expected sites. We calculated the absolute offset as the distance between the measured and expected nick-site positions. Across the analyzed nick sites, the offset was lower for SEM than for fluorescence microscopy, with values of 410 ± 325 bp and 1227 ± 820 bp, respectively (mean ± SD; Fig. 2C). The corresponding raw fractional contour positions and uncorrected contour-length distributions are shown in Supplementary Figs S5A and S6. Together, these results demonstrate that contour-based SEM positional readout enables accurate mapping of enzymatically introduced sequence-specific labels, establishing a foundation for extending this approach to direct protein–DNA complexes.

### SEM imaging of direct sequence-specific protein–DNA interactions

Having established in the nick-translation system that sequence-defined protein-associated labels can be visualized and used for positional readout in SEM, we next asked whether direct sequence-specific protein–DNA interactions could also be visualized in the same framework. In particular, the SA-eGFP label used above has a molecular weight of ~182 kDa, which is comparable to that of dCas9 (∼160 kDa), suggesting that dCas9 itself should also be capable of generating a detectable protein-associated signal in SEM. We therefore employed dCas9, which binds programmable target sequences, to test whether a densely bound sequence-specific protein region could be visualized directly on DNA and used for positional readout [51].

**Figure 3.**
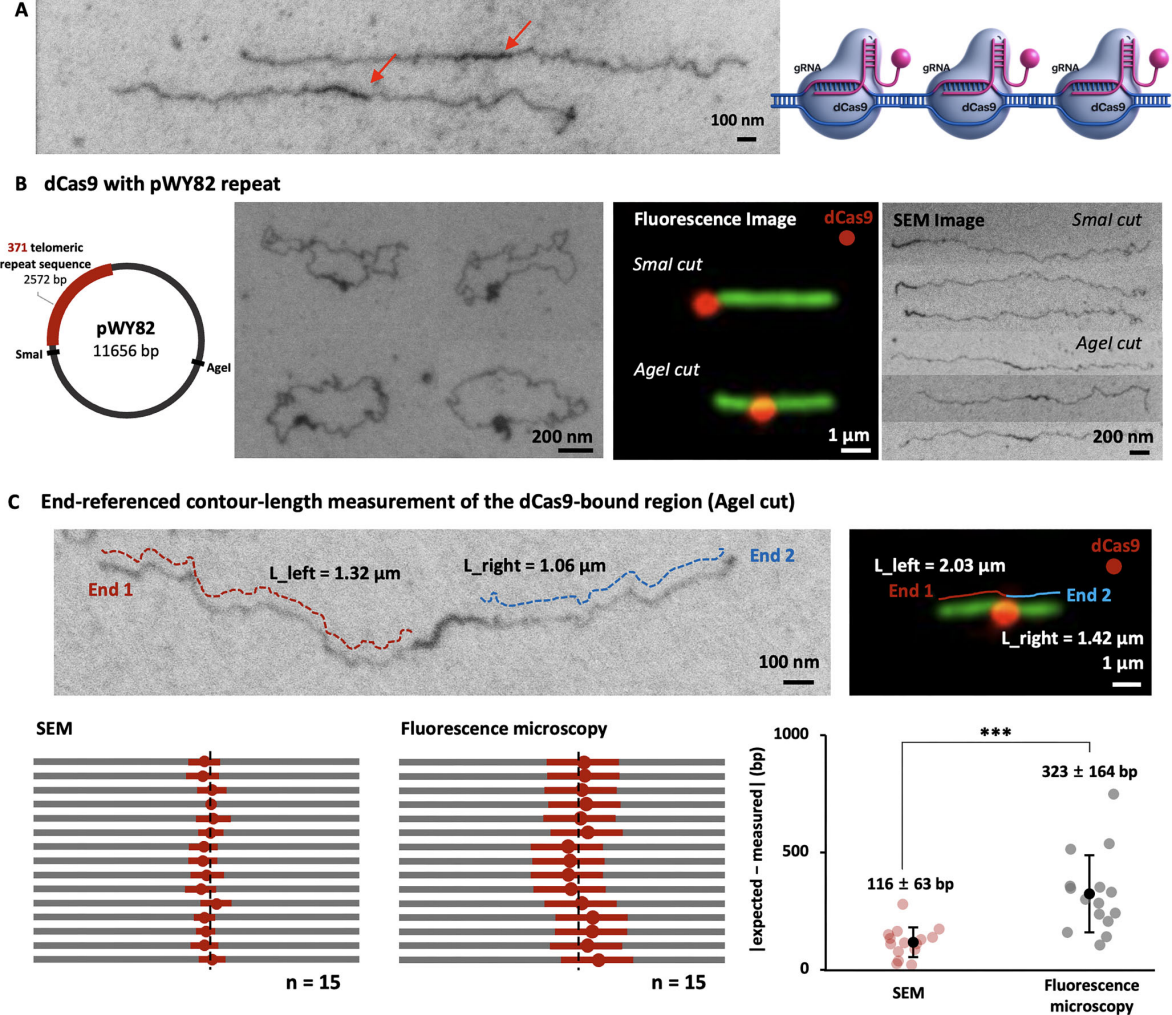
SEM imaging and positional readout of a densely dCas9-bound telomeric region. (**A**) Dense dCas9 binding on telomeric repeats in pWY82. SEM image of a linearized pWY82 molecule shows clearly darkened regions along the DNA contour, consistent with dense dCas9 binding within the repeat-rich segment. Arrows indicate representative darkened regions, and the schematic illustrates a dCas9–crRNA –Atto647-tracrRNA complex bound to its target sequence. (**B**) dCas9 binding on the telomeric repeat region in pWY82. The 11.6-kb plasmid contains a 2.6-kb array of maize telomeric repeats, providing a dense target region for sequence-specific dCas9 binding. Representative SEM images of circular pWY82 molecules are shown together with fluorescence and SEM images after linearization by digestion at the indicated restriction sites. The dCas9-bound region appears near one end after SmaI cut and near the middle after AgeI cut, consistent with the expected position of the repeat-rich segment. (**C**) End-referenced contour-length measurement and base-pair position readout of the dCas9-bound region. In the upper panel, contour distances from the darkened region to the two DNA ends were measured as L_left and L_right. The fluorescence image on the right shows the corresponding dCas9-labeled region after AgeI linearization. In the lower panel, positions of the darkened dCas9-bound region were read out in base-pair-equivalent coordinates by first calculating raw fractional contour positions from the measured end-referenced contour lengths and then converting these values using the corresponding reference length. Positions were compared across 15 SEM-imaged molecules and 15 fluorescence-imaged molecules. *n* = 15 was chosen to provide ∼95% statistical power to detect the observed effect size at α = 0.05. Each horizontal line represents an individual DNA molecule, highlighted segments indicate the dCas9-bound region, the vertical dashed line indicates the expected position, and circles mark the measured position per molecule. The plot on the right compares the absolute offsets between the measured and expected positions for SEM and fluorescence microscopy. Points and error bars indicate mean ± SD of absolute offsets. Welch’s *t*-test. ***P < .001.

We examined the 11.6 kb plasmid pWY82, which contains a 2.6 kb array of the maize telomere motif (TTTAGGG; 371 repeats) [52], providing a dense target region for sequence-specific dCas9 binding (Fig. 3A and B). Because this repeat-rich region contains many PAM-compatible target sites, we designed a guide RNA (GGTTTAGGGTTTAGGGTTTA) to drive dense sequence-specific binding within the telomeric array. Atto647-labeled tracrRNA was used for fluorescence visualization. SEM imaging of linearized and circular pWY82 molecules revealed distinct darkened regions along the DNA contour, consistent with dense dCas9 binding within the repeat-rich segment. After linearization by SmaI or AgeI digestion, the darkened region appeared near one DNA end or near the middle, respectively, matching the expected location of the repeat array.

Having identified densely dCas9-bound segments along DNA molecules, we next analyzed their positions relative to the DNA ends. For this end-referenced analysis, we measured the contour distances from the darkened region to the corresponding DNA ends, rather than using the measured length of the darkened segment itself (Fig. 3C, upper). The observed dark segment was substantially shorter than the nominal span of the targeted repeat region, with a mean measured length of 283 ± 74 nm (*n* = 15; CV ≈ 26%). This corresponds to an apparent shortening of ~32% relative to the expected nominal span. The substantial molecule-to-molecule variation in apparent dark-segment length further indicates that the densely bound repeat region did not follow a uniform stretching behavior and could not itself serve as a reliable positional marker, possibly due to local compaction or clustering of dCas9 complexes within the repeat-rich segment. We therefore represented the darkened region by a single representative point and calculated its position from the contour lengths of the flanking DNA on each molecule.

When the representative position of the darkened region was read out in base-pair-equivalent coordinates, SEM measurements clustered closer to the expected position than fluorescence (Fig. 3C, lower). To account for molecule-to-molecule differences in total contour length, raw fractional contour positions were first calculated from the measured flanking contour lengths and then converted to bp-equivalent coordinates using the corresponding flanking-reference DNA length. In SEM, the representative point of the dCas9-rich region was defined from the resolved darkened segment, whereas in fluorescence microscopy the position was defined by the centroid of the diffraction-limited dCas9 signal. The raw fractional contour positions and corresponding uncorrected contour-length distributions and stretching factors are shown in Supplementary Figs S5B and S7. For comparison across molecules, the representative point of the dCas9-rich region was used for positional analysis, while the full measured extent of the region, converted using the same stretching ratio, was retained separately for visualization (Fig. 3C, lower left). Across 15 molecules, SEM showed a smaller offset than fluorescence microscopy, with values of 116 ± 63 bp and 323 ± 164 bp, respectively (mean ± SD of absolute offsets; Fig. 3C, lower right). These results indicate that UranyLess/PVP-based SEM enables direct visualization of a densely dCas9-bound telomeric region and supports base-pair-referenced positional readout with improved positional accuracy and precision compared with fluorescence imaging.

### SEM visualization of sparse dCas9 binding on genomic DNA

**Figure 4.**
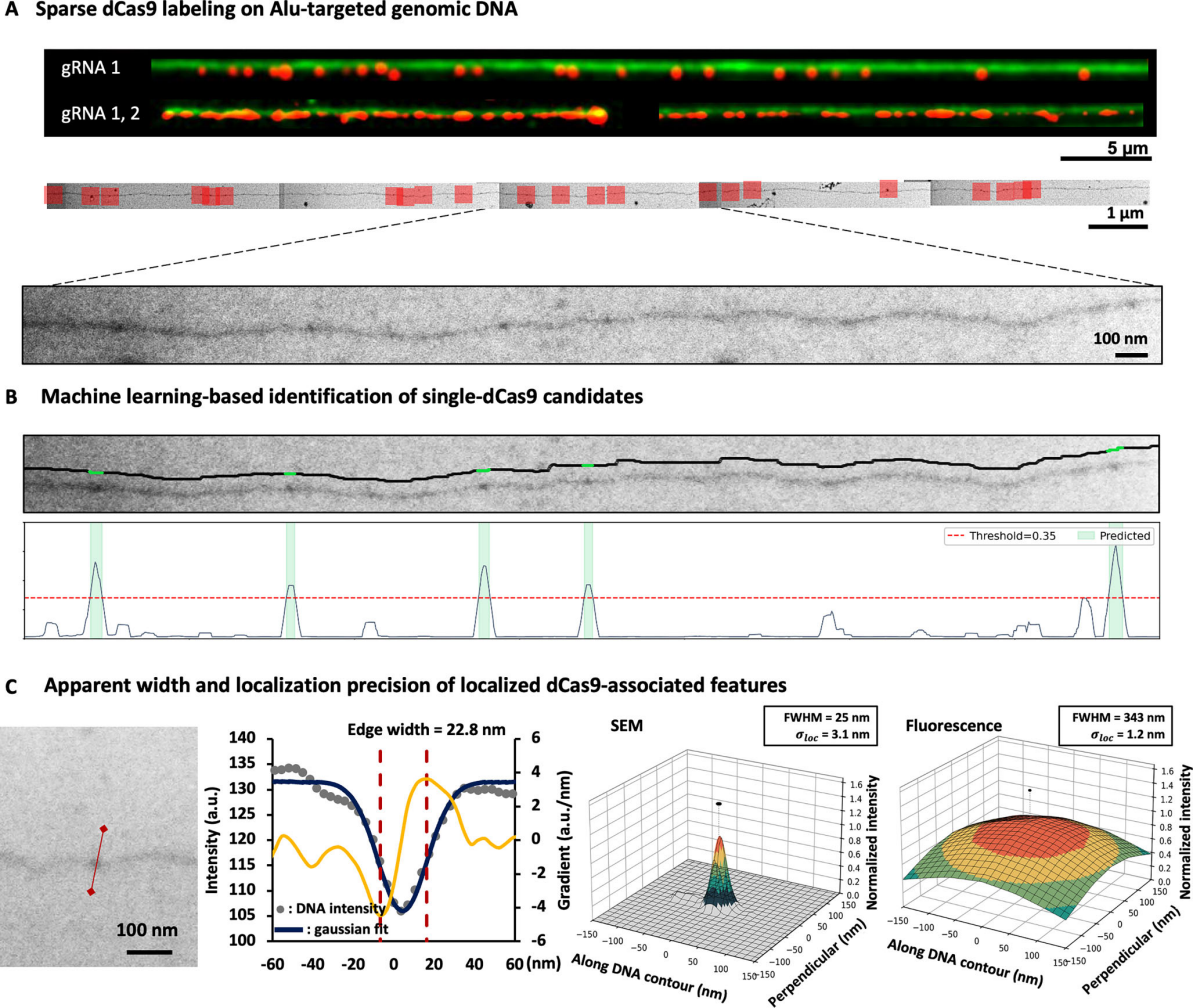
Identification, apparent width, and localization precision of localized dCas9-associated features on Alu-targeted genomic DNA. (**A**) Sparse Alu-targeted dCas9 labeling with Atto647-tracrRNA on human genomic DNA visualized by fluorescence microscopy and SEM at different magnifications. Representative fluorescence images obtained with gRNA1 and with gRNA1, 2 are shown at the top. Shaded boxes indicate SEM regions selected to match the fluorescence imaging scale for representative comparison. Representative SEM enlargements are shown below. (**B**) Machine-learning-based identification of localized dCas9-associated features. A representative traced DNA molecule is shown together with localized feature positions predicted along the contour. The lower plot shows the predicted probability of localized feature presence as a function of contour position, together with the classification threshold and predicted feature positions. (**C**) Apparent width and localization precision of localized dCas9-associated features. Left, SEM image of an individual localized feature. Middle, transverse intensity profile used to estimate the gradient-based apparent edge width of the localized SEM feature. Right, center-registered averaged SEM and fluorescence feature profiles used to estimate Gaussian FWHM and center-localization precision (σloc). SEM showed a much narrower apparent feature width than fluorescence microscopy, with FWHM values of 25.3 ± 4.0 nm and 342.7 ± 31.8 nm, respectively. In contrast, fluorescence microscopy showed smaller σloc for isolated foci than SEM, with values of 1.17 ± 0.10 nm and 3.08 ± 1.06 nm, respectively. Values indicate mean ± SD.

We next applied dCas9 labeling to Alu-targeted human genomic DNA to test whether sparse sequence-specific binding events could be resolved in SEM as localized features. Because the human genome contains over one million Alu elements (∼300 bp each) [53], this system was expected to generate multiple sparse binding events along individual genomic DNA molecules. To do this, we used two Alu-targeting guide RNAs (gRNA1 and gRNA2) and compared the resulting fluorescence and SEM images. In fluorescence microscopy, the labeled regions with Atto647-tracrRNA appeared as broader merged signals, whereas representative SEM images revealed discrete localized dCas9-associated features along the DNA contour (Fig. 4A). The red boxes in Fig. 4A indicate SEM regions selected to match the fluorescence imaging scale for representative comparison.

To identify these localized dCas9-associated features more systematically, we applied a machine-learning-based contour analysis to longitudinal intensity profiles extracted along traced DNA backbones (Fig. 4B). The model was trained on negative profiles from protein-free DNA and positive profiles from uniformly FP-DBP-coated DNA, together with additional SEM images of Alu-targeted dCas9-labeled genomic DNA. Using the calibrated random-forest classifier described in “Materials and methods” section, this approach enabled contour-based prediction of localized protein-associated features along individual DNA molecules. The profile features used in this analysis are summarized in Supplementary Fig. S8, and representative prediction outputs are shown in Fig. 4B, with additional examples in Supplementary Figs S9–S12.

These localized dCas9-associated features were then quantified by apparent feature width and center-localization precision (Fig. 4C). Candidate localized dCas9-associated features were detected along traced DNA contours (Fig. 4B and Supplementary Figs S6–S9), and the apparent widths of these localized features were measured from transverse intensity profiles in SEM and compared with fluorescence spot widths obtained by Gaussian fitting (Fig. 4C). SEM showed a much narrower apparent protein-associated feature profile than fluorescence microscopy, with FWHM values of 25.3 ± 4.0 nm and 342.7 ± 31.8 nm, respectively (mean ± SD). When center-localization precision was estimated from the fitted Gaussian center uncertainty, which incorporates both signal width and signal-to-noise ratio, fluorescence microscopy showed smaller σloc for isolated foci than SEM, with values of 1.17 ± 0.10 nm and 3.08 ± 1.06 nm, respectively. Together, these results show that SEM retains few-nanometer-scale center-localization precision while providing a much narrower apparent feature width, allowing closely spaced or dense protein-associated features to be spatially distinguished in a single SEM image.

### Comparison of SEM DNA imaging with TEM, AFM, and fluorescence microscopy

**Figure 5.**
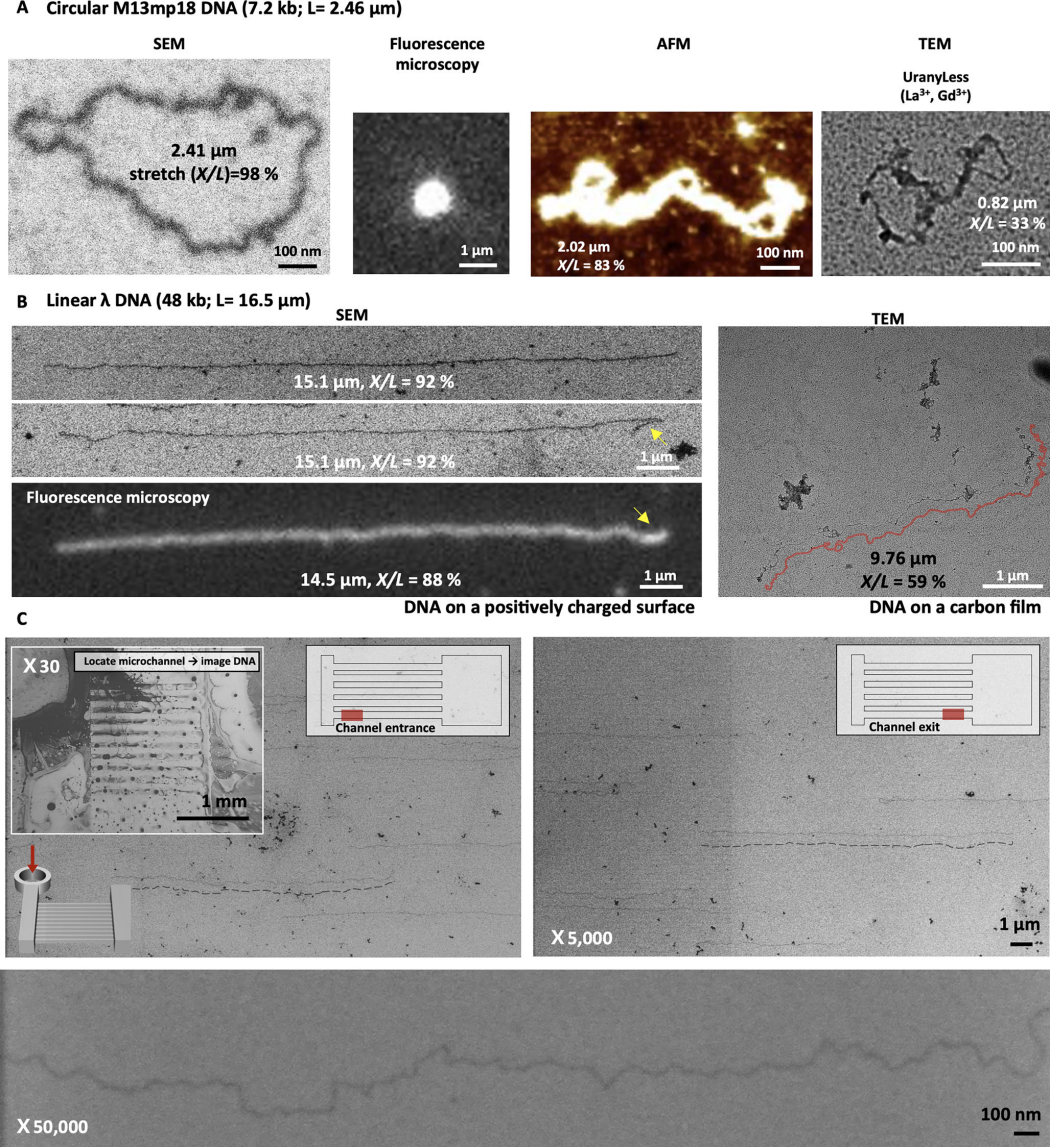
Comparison of SEM DNA imaging with fluorescence microscopy, AFM, and TEM. (**A**) SEM, fluorescence microscopy, AFM, and TEM images of circular M13mp18 RF1 DNA (7.2 kb). The stretch ratio (X/L) was defined as the apparent contour length (**X**) divided by the expected contour length (L). (**B**) SEM, fluorescence microscopy, and TEM images of long λ DNA (48.5 kb). For SEM and fluorescence microscopy, λ DNA was stretched on a positively charged surface using a microfluidic device. For TEM, λ DNA was deposited on a carbon film by a drag-and-dry method and then stained with UranyLess. (**C**) SEM visualization of DNA molecules on a positively charged silicon surface. Low-magnification imaging (×30) enables localization of the microchannel array, followed by imaging at ×5000 near the channel entrance and exit. Boxes in the schematics indicate the imaged regions. The bottom panel (×50 000) shows a high-resolution view of an individual DNA molecule.

Figure 5 compares SEM DNA imaging using UranyLess and PVP with fluorescence microscopy, AFM, and TEM. Across these comparisons, SEM provides clear DNA contrast with directly interpretable single-molecule morphology under our experimental conditions. Importantly, sample preparation methods commonly used for fluorescence microscopy, including charged-surface deposition and microfluidic stretching, can be readily adapted for SEM imaging, enabling DNA manipulation and visualization from low to high magnification with minimal sample input. A summary of scan area, acquisition time, and apparent DNA width across imaging modalities is provided in Supplementary Fig. S13 [9, 11, 15, 54–57].

We first compared the four modalities using circular double-stranded DNA (M13mp18 RF1, 7.2 kb; 7249 bp) (Fig. 5A). SEM revealed a well-stretched molecule with clear contrast, with a measured contour length of 2.41 μm (∼98% of the theoretical length, 7249 bp × 0.34 nm/bp). In contrast, fluorescence microscopy produced a diffraction-limited bright spot (∼1.15 μm diameter) without resolvable structural information. In our comparison experiment, AFM imaging of the same construct revealed a supercoiled circular structure with a stretch ratio of ∼83% (X/L), highlighting its strength for topographic visualization of DNA topology. Although optimized AFM conditions can provide highly accurate contour-length and protein-position measurements, the relatively small scan area and iterative search process [14, 54, 56, 58] under our sparse-molecule comparison conditions made sparsely distributed molecules more time-consuming to identify and image. In contrast, SEM enabled acquisition of an isolated molecule within tens of seconds, whereas AFM required substantially longer search and acquisition times. HS-AFM can capture DNA dynamics under optimized conditions [15, 59, 60], but such measurements remain sensitive to surface chemistry, ionic conditions, and imaging parameters.

We next compared SEM with TEM using the same circular DNA substrate (Fig. 5A). TEM can provide high spatial resolution, but successful visualization of extended DNA molecules is highly sensitive to grid preparation and spreading conditions [61, 62]. In our workflow, DNA was immobilized on a carbon film before UranyLess staining [20] to minimize aggregation or condensation caused by multivalent cations [19]. Even so, substantial shrinkage was observed during drying, consistent with weak interactions between DNA and the carbon surface. As a result, the TEM image in Fig. 5A showed only 33% stretch (X/L) for the 7.2 kb circular DNA, markedly lower than the values obtained by SEM (98%) or AFM (83%).

Because shrinkage can be more pronounced for circular DNA, we also examined long linear λ DNA (48.5 kb) (Fig. 5B). For SEM and fluorescence microscopy, λ DNA molecules were elongated on positively charged surfaces using a microfluidic device, yielding 92% and 88% of the expected contour length, respectively. The same microchannel-assisted workflow also supported practical low-input SEM imaging over a large field of view, allowing stretched DNA molecules to be identified at low magnification and then imaged at higher magnification (Fig. 5C). However, this approach was not directly transferable to our TEM workflow. Instead, λ DNA was deposited onto carbon films by a drag-and-dry method and then stained with UranyLess. Under these conditions, many DNA molecules in TEM image appeared aggregated, although one molecule reached ∼59% stretch when curvature and twisting were considered. Notably, an early TEM study in 1964 reported stretched λ DNA with a measured length of 17.2 μm [63], indicating that substantially extended TEM images can be obtained under appropriate conditions. In our setup, however, we were unable to reproduce comparable stretching. By contrast, SEM consistently produced well-extended λ DNA with clear contrast and resolved sub-100 nm structural details, including the kink indicated by the yellow arrow.

## Discussion

In this study, we established an SEM-based mapping platform for sequence-specific protein–DNA interactions on individual long DNA molecules. By combining the heavy-metal reagent UranyLess with our previously developed protein–polymer overlayer [27, 28], we obtained robust DNA contrast on silicon wafer and, on the same molecules, simultaneously detected protein-bound regions as locally modulated SEM features. Tuning the polymer concentration further modulated the relative visibility of protein-associated regions, providing a practical means to bias the contrast toward protein detection without compromising backbone visualization.

The physical basis for these measurements distinguishes SEM from optical single-molecule imaging. Fluorescence-based approaches infer position from diffraction-broadened intensity centroids and therefore measure an effective end-to-end distance modulated by the point-spread function, whereas SEM directly traces the DNA contour at sub-nanometer pixel scales. For molecules elongated to 92%–98% of the theoretical B-form contour length (0.34 nm/bp) on functionalized silicon, the traced path closely approximates the polymer-physical contour length. This near-equivalence between traced and theoretical contour, together with molecule-specific stretching correction, provides the geometric foundation for base-pair-referenced positional readout demonstrated in Figs 2 and 3.

Within this framework, three distinct binding regimes were resolved on individual molecules. Nick-translated SA-FP-labeled λ DNA generated short darkened tracts whose end-referenced positions yielded a mean offset of 410 ± 325 bp from the expected nick site, ~3-fold lower than the centroid-based offset obtained by fluorescence microscopy (1227 ± 820 bp; Fig. 2C). Dense dCas9 binding on the maize-telomere repeat array of pWY82 produced a continuous dark segment whose representative position fell within 116 ± 63 bp of the expected coordinate, again ~3-fold tighter than the corresponding fluorescence centroid (323 ± 164 bp; Fig. 3C). Finally, sparse Alu-targeted dCas9 binding on human genomic DNA produced discrete localized features along the DNA contour, which were systematically detected by a calibrated random-forest classifier with out-of-fold performance of F1 = 0.97 (precision = 0.97, recall = 0.98). These features exhibited a much narrower apparent width than diffraction-limited fluorescence spots, while the σloc analysis showed that center-localization uncertainty also depends on signal-to-noise ratio and was smaller for isolated fluorescence foci than SEM features (Fig. 4C). Together, these three regimes show that the same contrast principle scales from sequence-defined enzymatic labels to direct, base-pair-referenced readout of natively bound sequence-specific complexes.

Compared with the dominant single-molecule DNA imaging modalities, this SEM workflow occupies a distinct operating point. Relative to TEM (Fig. 5), planar silicon substrates supported substantially better DNA elongation (98% versus 33% stretch for circular M13mp18; 92% versus ∼59% for λ DNA) and were directly compatible with the microfluidic stretching procedures developed for fluorescence imaging. Relative to AFM, SEM enabled rapid identification of sparse molecules across multi-scale magnifications (×10 to > ×100 000 on the same instrument) without iterative tip-based search, an advantage that becomes critical when targets are rare in the field. Relative to fluorescence imaging, SEM trades multicolor multiplexing for a three-fold improvement in positional accuracy and precision and for direct contour visualization. As a consequence, this approach is most naturally complementary to existing optical genome mapping platforms such as Bionano [64], where SEM can verify and re-localize labeled landmarks at base-pair precision on the same nick-labeling chemistry already in widespread use.

Several limitations frame the present implementation. First, our approach requires immobilization, fixation, and dehydration on functionalized silicon, and therefore reports a single static snapshot per molecule rather than the binding dynamics accessible by HS-AFM [15] or DNA-curtain fluorescence [7]. Second, single-channel secondary-electron contrast does not directly support multicolor multiplexing across protein species; protein-specific identification will require complementary strategies such as nanoparticle-tagged antibodies or electron-dense reporters of distinguishable size or composition. Third, densely bound protein clusters appear as continuous dark segments whose internal binding events cannot be individually resolved—a property reflecting local clustering rather than a fundamental optical limit, as evidenced by the discrete features observed for sparse dCas9 binding on genomic DNA. Finally, the current sample sizes (*n* = 15 molecules per modality in Figs 2C and 3C; *n* = 32 features in Fig 4C) are modest; while sufficient to establish the reported effect sizes, larger cohorts will be required for genome-wide mapping applications.

These limitations are largely addressable through routine engineering rather than requiring fundamental advances. Continued surface engineering, standardized sample handling, and automated image analysis should expand throughput, and integration with electron-dense protein-specific labels should enable orthogonal multiplexing. More broadly, the contrast behavior described here suggests adaptability to a wide range of sequence-specific DNA-binding proteins, including transcription factors, restriction enzymes, and other programmable DNA-targeting systems. Such generality opens concrete application opportunities: direct visualization of chromatin organization and nucleosome positioning on individual long DNA molecules; targeted genome-mapping workflows for clinical diagnostics, including identification of pathogenic alleles in repetitive or rearranged regions; and single-molecule detection of structural variations such as insertions, deletions, and complex rearrangements that are difficult to resolve by short-read sequencing alone. By providing direct, base-pair-referenced positional readout on individual long DNA molecules, this SEM framework complements and extends fluorescence-based optical genome mapping platforms and offers a practical path toward high-resolution single-molecule mapping of protein–DNA interactions across the genome.

## Supplementary Material

gkag687_Supplemental_Files

## Data Availability

All data supporting the findings of this study are available within the article and its Supplementary Information. The analysis code is publicly available at https://github.com/jolaboffice/SEM_DNA_Analysis and https://doi.org/10.5281/zenodo.20827106. Additional SEM/TEM images are available from the corresponding author upon reasonable request.
