# Supplementary material for: SEM mapping of sequence-specific protein–DNA interactions on long DNA molecules
Source: Nucleic Acids Res. 2026 Jul 9;54(13):gkag687. doi: 10.1093/nar/gkag687 (PMC13347267; doi:10.1093/nar/gkag687)
Supplement: gkag687_Supplemental_Files [file gkag687_supplemental_files.zip › SI0604.docx]

**SEM Mapping of Sequence-Specific Protein–DNA Interactions on Long DNA Molecules**

Chanyoung Noh^1†^, Sangwon Lee^1†^, Yoonjung Kang^1†^, Taesoo Kim^1^, Taebin Yun^1^, Yoojin Kim^1^, Gyuri Park^1^, Priyannth R. Sundharbaabu^2^, Sang-Hee Shim^3^, Kwang-il Lim^4^*, Jung Heon Lee^2,5^*, Kyubong Jo^1,6^*

^1^Department of Chemistry, Sogang University; Seoul 04107, Korea

^2^School of Advanced Materials Science and Engineering, Sungkyunkwan University (SKKU); Suwon 16419, Korea

^3^Department of Chemistry, Korea University, Seoul, 02841 Korea

^4^Department of Chemical and Biological Engineering, Sookmyung Women's University, Seoul, 04312 Korea

^5^Department of MetaBioHealth, Sungkyunkwan University (SKKU); Suwon 16419, Korea.

^6^Center for Nano Materials, Sogang University; Seoul 04107, Korea

* To whom correspondence should be addressed. Email: jokyubong@sogang.ac.kr
Correspondence may also be addressed to jhlee7@skku.edu and klim@sookmyung.ac.kr

**Supporting Information (SI)**

**Materials and Methods**

- **Chemicals**
- **FP-DBP (fluorescent protein–conjugated DNA-binding protein)**
  - Truncated transcription activator-like effector-mNeonGreen (tTALE-mNG)
- **SA-FP (Streptavidin–conjugated fluorescent protein)**
  - Streptavidin-RRvT
  - Streptavidin-eGFP
- **Protein expression and purification**
- **Preparation of microfluidic device and positively charged silicon wafer surface**
- **AFM DNA Imaging**
- **TEM DNA Imaging**
- **SEM DNA Imaging**
  - UranyLess with/without PVP
  - DNA binding protein–PVP
  - UranyLess and DNA binding protein–PVP
- **Sequence-specific protein-bound DNA molecules**
  - dCas9-labeled pWY82 DNA molecules
  - dCas9-labeled HG002 genomic DNA molecules
  - SA-FP labeled DNA molecules
- **DNA preparation from HG002 cells**
  - HG002 cell preparation
  - DNA preparation from HG002
- **Image analysis**

**Supplementary Figures**

- **SI Figure 1 : Characterization of UranyLess and comparison with alternative metal-staining conditions for SEM DNA imaging.**
- **SI Figure 2 : Workflow for DNA backbone tracing and contour-based intensity measurement in SEM images.**
- **SI Figure 3 : Gradient-based measurement of apparent DNA edge width from contour-aligned transverse profiles.**
- **SI Figure 4 : Site 7-8 gap analysis of nick-translated λ DNA labeled with SA-FP.**
- **SI Figure 5 : Raw fractional contour positions of sequence-labeled sites.**
- **SI Figure 6 : Uncorrected contour-length distributions and stretching factors of SA-FP labeled lambda DNA.**
- **SI Figure 7 : Uncorrected contour-length distributions and stretching factors for dCas9-bound segment analysis.**
- **SI Figure 8 : Machine learning features used for contour-based prediction of protein-associated signals.**
- **SI Figure 9 : Machine learning prediction outputs for negative-control DNA.**
- **SI Figure 10 : Machine learning prediction outputs for positive-control DNA.**
- **SI Figure 11 : Machine learning prediction outputs for Alu-targeted dCas9-bound genomic DNA.**
- **SI Figure 12 : Additional machine learning prediction outputs for Alu-targeted dCas9-bound genomic DNA.**
- **SI Figure 13 : Summary comparison of DNA imaging methods by acquisition time and apparent DNA width.**

**Supplementary Dataset. Raw quantitative data underlying Figures 1, 2, 3, and SI Figure 1. Provided as an Excel file.**

- **Contrast_SNR_measurements.xlsx : Fig. 1H, SI Fig. 1G,H**
- **Position_analysis.xlsx : Fig. 2B, Fig. 3C**

**Materials and Methods**

**Chemicals**

Polyvinylpyrrolidone (PVP) was purchased from Sigma-Aldrich (St. Louis, MO, USA). Quantum dot 585 streptavidin conjugate, SYTOX Deep Red were obtained from Thermo Fisher Scientific (Waltham, MA, USA). Oxidized silicon wafers were purchased from Wafer Market (Yong-In, Korea). N-Trimethoxysilylpropyl-N,N,N-trimethylammonium chloride was obtained from Gelest (Morrisville, PA, USA). Bacteriophage λ DNA (48.5 kb), M13mp18 double-stranded DNA (7.2 kb), Nb.BssSI, Proteinase K, β-Agarase I, and EnGen Spy dCas9 (SNAP-tag) were purchased from New England Biolabs (Ipswich, MA, USA). DNA polymerase I and RecA were obtained from Enzynomics (Daejeon, Korea). Biotin-16-dUTP and ATPγS were purchased from Jena Biosciences (Thuringia, Germany). RPMI 1640 medium and 1× antibiotic–antimycotic were purchased from Gibco (Waltham, MA, USA). UranyLess EM stain was obtained from Electron Microscopy Sciences (Hatfield, PA, USA). AMPure XP beads were purchased from Beckman Coulter (Indianapolis, IN, USA). The QIAprep Spin Miniprep Kit was purchased from Qiagen (Germantown, MD, USA). Alt-R CRISPR-Cas9 tracrRNA (Atto 647-labeled), Alt-R CRISPR-Cas9 crRNA, and nuclease-free duplex buffer were obtained from Integrated DNA Technologies (Coralville, IA, USA).

**FP-DBP (fluorescent protein–conjugated DNA-binding protein)**

**Truncated transcription activator-like effector-mNeonGreen (tTALE-mNG).** The tTALE-mNG plasmid (65 kDa) was prepared as previously described (1,2). The fluorescent protein was fused to the C-terminus of the truncated TALE domain using restriction–ligation cloning, and a flexible GGSGG linker was placed between the DNA-binding domain and mNeonGreen. The complete amino acid sequence is listed below:

MGSSHHHHHHSSGLVPRGSHMDLRTLGYSQQQQEKIKPKVRSTVAQHHEALVGHGFTHAHIVALSQHPAALGTVAVKYQDMIAALPEATHEAIVGVGKQWSGARALEALLTVAGELRGPPLQLDTGQLLKIAKRGGVTAVEAVHAWRNALTGAPLNLTPAQVVAIASNNGGKQALETVQRLLPVLCQDHGLTPAQVVAIASNGGGKQALETVQRLLPVLCQAHGLTPDQVVAIASHDGGKQALETVQRLLPVLCQDHGLTPAQVVAIASNGGGKQALETVQRLLPVLCQAHGLTPDQVVAIASNNGGNEQALETVQRLLPVLCQAHGLTPAQVVAIASNGGGKQALETVQRLLPVLCQDHG**GGSGG**PGMVSKGEEDNMASLPATHELHIFGSINGVDFDMVGQGTGNPNDGYEELNLKSTKGDLQFSPWILVPHIGYGFHQYLPYPDGMSPFQAAMVDGSGYQVHRTMQFEDGASLTVNYRYTYEGSHIKGEAQVKGTGFPADGPVMTNSLTAADWCRSKKTYPNDKTIISTFKWSYTTGNGKRYRSTARTTYTFAKPMAANYLKNQPMYVFRKTELKHSKTELNFKEWQKAFTDVMGMDELYKGSGC

**SA-FP (Streptavidin–conjugated fluorescent protein)**

**Streptavidin-RRvT.** The Streptavidin-RRvT plasmid (73.5 kDa) was prepared as previously described (3). The fluorescent protein was fused to the C-terminus of streptavidin via a GGSGG linker. The complete amino acid sequence is listed below:

MGSSHHHHHHSSGLVPRGSMASMTGGQQMGAGITGTWYNQLGSTFIVTAGADGALTGTYESAVGNAESRYVLTGRYDSAPATDGSGTALGWTVAWKNNYRNAHSATTWSGQYVGGAEARINTQWLLTSGTTEANAWKSTLVGHDTFTKVKPSAASIDAAKKAGVNNGNPLDAVQQ**GGSGG**PGMVSKGEEVIKEFMRFKVRMEGSMNGHEFEIEGEGEGRPYEGTQTAKLKVTKGGPLPFAWDILSPQFMYGSKAYVKHPADIPDYKKLSFPEGFKWERVMNFEDGGLVTVTQDSSLQDGTLIYNVKMRGTNFPPDGPVMQKKTMGWEASTERLYPRDGVLKGEIHQALKLKDGGHYLVEFKTIYMAKKPVQLPGYYYVDTKLDITSHNEDYTIVEQYERSEGRHHLFLYGMDELYKGSTGSGSSGPMVSKGEEAIKEFMRFKVSMEGSMNGHEFEIEGEGEGRPYEGTQTAKLKVTKGGPLPFAWDILSPQFMYGSKAYVKHPADIPDYKKLSFPEGFRWERVMNFEDGGLVTVTQDSSIQDGTLIYKVKVRGTNFPPDGPVMQKKTMGWEASTERLYPRDGVLKGEIHQALKLKDGGHYLVEFKTIYMAKKPVQLPGYYYVDTKLDITSHNEDYTVVEQYERSEGRHHLFLYGMDELYKGSGC

**Streptavidin-eGFP.** The Streptavidin–eGFP plasmid (45.5 kDa) was constructed using the same cloning strategy as Streptavidin–RRvT. The eGFP gene was PCR-amplified from pRSET-mSA2-eGFP (Addgene #39862) using the following primers:
Forward: 5′-CGGCTCTGGCGGCCCCGGGGTGAGCAAGGGCGAGGAGC-3′
Reverse: 5′-GCTTTGTTAGCAGCCGGATCCTTACTTGTACAGCTCGTCC-3′
The amplified eGFP fragment was fused to streptavidin through a GGSGG linker. The complete amino acid sequence is listed below:

MGSSHHHHHHSSGLVPRGSMASMTGGQQMGAGITGTWYNQLGSTFIVTAGADGALTGTYESAVGNAESRYVLTGRYDSAPATDGSGTALGWTVAWKNNYRNAHSATTWSGQYVGGAEARINTQWLLTSGTTEANAWKSTLVGHDTFTKVKPSAASIDAAKKAGVNNGNPLDAVQQ**GGSGG**PGVSKGEELFTGVVPILVELDGDVNGHKFSVSGEGEGDATYGKLTLKFICTTGKLPVPWPTLVTTLTYGVQCFSRYPDHMKQHDFFKSAMPEGYVQERTIFFKDDGNYKTRAEVKFEGDTLVNRIELKGIDFKEDGNILGHKLEYNYNSHNVYIMADKQKNGIKVNFKIRHNIEDGSVQLADHYQQNTPIGDGPVLLPDNHYLSTQSALSKDPNEKRDHMVLLEFVTAAGITLGMDELYK

**Protein expression and purification**

The constructed plasmids were transformed into *E. coli* BL21 (DE3) strains using a standard cloning technique. The BL21 cells carrying the FP-DBP plasmid were cultured in a Luria Broth (LB) medium containing ampicillin at 37°C for overnight (16 hr). Afterward, 1 mL of the overnight culture was transferred to 100 mL of fresh LB medium with ampicillin and incubated at 37°C until the optical density at 600 nm (OD600) reached 0.4–0.6. Protein expression was induced by adding 1 mM IPTG, followed by incubation at 20–25°C with shaking at 200 rpm for 16 hr overnight. The cells were then subjected to ultrasonication for 15 min and centrifuged for 10 min at 10,000 rpm. The FP-DBP protein was purified by affinity chromatography using Ni-NTA agarose resin. Elution was carried out with a buffer containing 50 mM Na_2_HPO_4_, 300 mM NaCl, and 250 mM imidazole at pH 8.0. The purified protein was buffer exchanged with 1×TE buffer (Tris 10 mM and EDTA 1 mM, pH 8.0) and stored at -20°C with the addition of glycerol.

**Preparation of microfluidic device and positively charged silicon wafer surface**

Polydimethylsiloxane (PDMS) microchannel was made as previously described (4,5), with device dimensions of 100 µm in width and 2.4 µm in height. The method to make positively charged glass surface was adapted to make positively charged silicon wafers (6,7). Silicon wafers with a 30-nm SiO_2_ layer were obtained from Wafer Market (Yong-In, Korea). The wafers were incubated in a 250-mL solution containing N-trimethoxysilylpropyl-N,N,N-trimethylammonium chloride (1.1 mM) at 65°C with agitation at 100 rpm for 16 hrs. Afterward, they were washed three times with 99.9% ethanol and stored in 99.9% ethanol.

**TEM DNA Imaging**

A 10 µL droplet of DNA solution was placed on a parafilm and contacted with a carbon-coated TEM grid for 1 min. After drying, the grid was placed on a droplet of UranyLess stain for 1 min, then dried again.

**SEM DNA Imaging**

DNA samples diluted in 1× TE were loaded (1 µL) into PDMS microchannels on positively charged silicon wafers. After drying for 50 min, the PDMS device was removed. SEM imaging was performed on an Apreo 2S HiVac microscope at accelerating voltages of 10 kV and images were processed using ImageJ.

**1. UranyLess:**
After DNA loading, 0.5 µL of UranyLess was loaded onto the wafer after 5 min.

**2. DNA binding protein–PVP:**
DNA was incubated with tTALE-mNG for 10 min, mixed 1:1 with 5% PVP (40 kDa), and then loaded.

**3. UranyLess and PVP:**
After DNA loading, 0.5 µL of UranyLess followed by 0.5 µL of PVP was added with 5-min intervals.

**4. UranyLess and DNA binding protein–PVP:**
DNA was incubated with tTALE-mNG for 10 min, loaded, then 0.5 µL UranyLess and 0.5 µL of PVP were applied sequentially.

**Sequence-specific protein bound DNA molecules**

**dCas9 labeled pWY82 DNA molecules.** Guide RNAs (gRNAs) were prepared by mixing 1 μL of 100 μM Atto647-labeled tracrRNA, 1 μL of crRNA (GGTTTAGGGTTTAGGGTTTA), 1.6 μL of DEPC-treated water, and 0.4 μL of Nuclease-Free Duplex Buffer (IDT). The mixture was incubated at 95 °C for 5 min and then at 25 °C for 10 min to allow annealing. The annealed gRNA was diluted with 96 μL of DEPC-treated water to a final concentration of 1 μM. For ribonucleoprotein (RNP) assembly, equal volumes of 1 μM gRNA and 1 μM SNAP-dCas9 were mixed and incubated at room temperature for 10 min. DNA binding was performed in a 20 μL reaction containing 50 ng of pWY82 plasmid DNA, 6.3 μL of gRNA–dCas9 RNP, 2 μL of 10× NEBuffer 3.1, and DEPC-treated water, followed by incubation at 37 °C for 1 hr.

**dCas9 labeled HG002 genomic DNA molecules.** Two crRNAs (CTGCACTCCAGCCTGGGCGA and CACTTGAACCCAGGAGGCAG) were each annealed with Atto647-labeled tracrRNA under the same conditions and diluted with 96 μL of DEPC-treated water to yield 1 μM gRNAs. The two gRNAs were mixed at a molar ratio of 7:1 prior to RNP assembly. For RNP formation, equal volumes of the mixed gRNAs and 1 μM SNAP-dCas9 were combined and incubated at room temperature for 10 min. DNA binding was performed in a 10 μL reaction containing 5 ng of HG002 genomic DNA, 2.5 μL of gRNA–dCas9 RNP, and 1 μL of 10× NEBuffer 3.1, followed by incubation at 37 °C for 1 hr.

**SA-FP labeled DNA molecules.** SA-FP labeling was carried out after nick translation using nickase (Nb.BssSI) treatment. A total of 500 ng of λ DNA was incubated with 20 units of Nb.BssSI and 10 units of DNA polymerase I in 1× DNA Polymerase I Buffer at 16°C for 2 hrs. The labeling mixture included biotin-labeled dUTP (1.1 µM), dTTP (5.5 µM), dATP (5.5 µM), dCTP (5.5 µM), and dGTP (5.5 µM). After nick translation, the DNA solution was dialyzed using a drop dialysis protocol with a 25-nm mixed cellulose ester membrane. The recovered solution was diluted to a final concentration of 2 ng/µL. This solution was then mixed with 150 nM of streptavidin-RRvT and incubated at 20–25°C for 10 min.

**DNA preparation from HG002 cell**

**HG002 cell preparation.** HG002 (GM24385) cells were obtained from Coriell Institute. The cells were cultured in RPMI 1640 medium supplemented with a final concentration of 15% fetal bovine serum and 1× antibiotic-antimycotic. Once the cells reached approximately 80% confluence in the culture flask, the entire solution (10 mL) was transferred into a 15-mL conical tube and centrifuged at 1000 × g for 5 min. The medium was then carefully removed using a Pasteur pipette. The HG002 cells were resuspended in 1 mL of 1× PBS and transferred into an Eppendorf tube. After two washes with 1× PBS buffer, the cells were resuspended in 500 μL of 1× PBS buffer.

**DNA preparation from HG002.** DNA was extracted using agarose gel plugs. The cell solution was mixed with a 2% 1× TE Low Gelling Temperature (LGT) agarose solution to achieve a final LGT concentration of 0.7%, and 20 μL aliquots were prepared, followed by incubation at 4°C for 1 hr. Proteinase K was added to a final concentration of 2 mg/mL, and the mixture was incubated at 42°C for 4 hrs. An additional equal amount of proteinase K was added, followed by overnight incubation at 42°C for 16 hrs. The agarose plugs were washed four times with 1× TE buffer for 30 min each. 1 μL of β-Agarase I was added after melting the agarose plugs at 65°C for 20 min. Finally, the DNA solution was prepared by incubation at 42°C for 1 hr, followed by heat inactivation of β-Agarase I at 65°C for 20 min.

**Image analysis.** DNA backbone contours in SEM and TEM images were traced using a dynamic-programming algorithm applied to CLAHE-enhanced and Meijering-filtered line-strength maps, and the trace with the higher mean dark-path intensity was selected automatically. At each trace point, a 41-pixel intensity profile was extracted perpendicular to the local tangent. For direct comparison across magnifications, per-point profiles were resampled onto a common nm grid (±65 nm, 41 samples). Apparent DNA edge-to-edge width was measured on the per-run averaged profile by sub-pixel localization of the intensity-gradient extrema.

For machine learning-based detection of protein-associated signals, each profile was summarized by 82 scale-invariant features derived from the edge-baseline-normalized trace, including multi-scale central-window intensity descriptors, first- and second-derivative features, peak-relative widths, left/right asymmetry, and statistical moments. A calibrated random-forest classifier with sigmoid calibration was trained on 13,147 profiles (3,551 positive and 9,596 negative) from 13 manually annotated images, using 600 trees, balanced-subsample class weighting, and 5-fold internal cross-validation. Leave-one-positive-image-out cross-validation was used to prevent data leakage and to evaluate out-of-fold probabilities. The along-trace Gaussian smoothing window, minimum run-length filter, and decision threshold were jointly grid-searched on the cross-validation probabilities to maximize F1 score, yielding a final setting of approximately 38 nm smoothing, 24 nm minimum run length, and a decision threshold of 0.35. Out-of-fold performance was F1 = 0.97, with precision = 0.97 and recall = 0.98.

Protein localization precision (σ_loc) was estimated as the standard error of the fitted Gaussian center derived from the fit covariance, providing a covariance-based estimate of localization uncertainty. Per-pixel noise was estimated from signal-free regions: unbound-backbone scatter for SEM and image background for fluorescence microscopy. Thus, σ_loc depends on both the fitted signal width and the local noise level, reflecting the combined effect of signal width and signal-to-noise ratio. The apparent width was defined as the FWHM of the average protein-associated feature profile after registering features to a common sub-pixel center. For fluorescence microscopy, only spatially isolated foci with no neighboring focus within ~2× the PSF width were retained (n = 50 foci from three single-gRNA images). For SEM (n = 32 segments from 10 SEM images), the bare-DNA cross-section was subtracted so that the measured peak reflected the bound-protein-associated contribution. Full implementation and all analysis code are available on GitHub.

**Supplementary Figures**


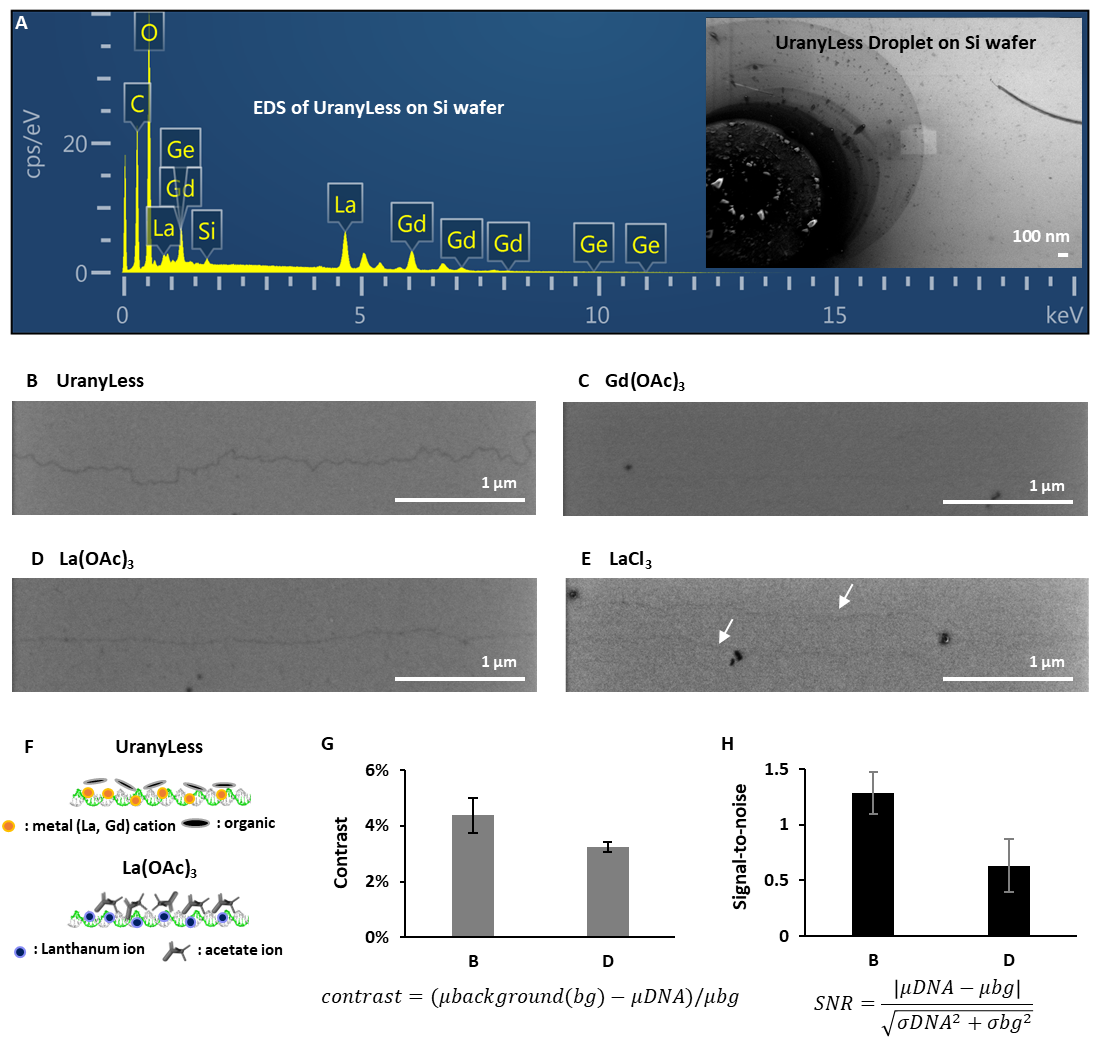


**Figure S1. Characterization of UranyLess and comparison with alternative metal-staining conditions for SEM DNA imaging. (A)** Energy-dispersive spectroscopy (EDS) spectrum of UranyLess on Si wafer, showing La, Gd, Ge, and prominent carbon and oxygen signals, indicative of organic components. Inset: SEM image of a UranyLess droplet on Si wafer. **(B)** SEM image of DNA stained with UranyLess alone. **(C)** SEM image of DNA treated with gadolinium acetate [Gd(OAc)₃], showing no detectable DNA features. **(D)** SEM image of DNA treated with lanthanum acetate [La(OAc)₃], showing visible DNA strands. **(E)** SEM image of DNA treated with LaCl₃, showing faint DNA signals (white arrows). **(F)** Schematic illustration of possible coordination of UranyLess or La(OAc)₃ with DNA. (G) Quantitative comparison of Weber contrast for clearly distinguishable DNA molecules under conditions (B) and (D). (H) Quantitative comparison of detectability (SNR) under conditions (B) and (D). For each condition, three independent images were analyzed, and the error bars represent the standard deviation among images.


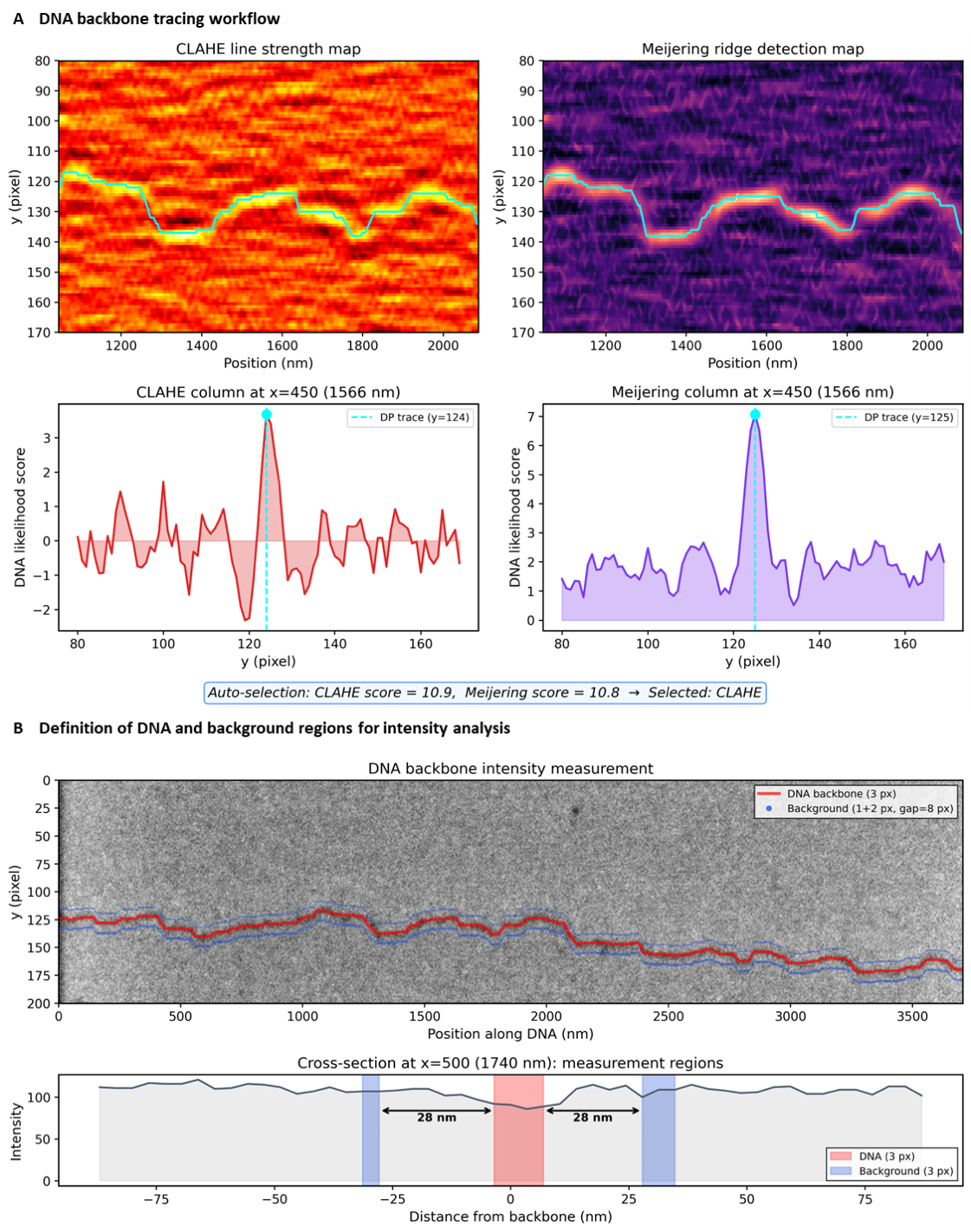


Figure S2. Workflow for DNA backbone tracing and contour-based intensity measurement in SEM images. (A) Representative example of DNA backbone tracing using two candidate line-strength maps generated from the same SEM image region. CLAHE-based enhancement and Meijering ridge detection were used to generate candidate traces, and column-wise trace scores at a representative x-position are shown below. The final DNA backbone trace was selected from the higher-confidence candidate.
(B) Definition of DNA and background regions for contour-based intensity analysis. DNA-associated intensity was measured along the traced backbone, while background intensity was measured from flanking regions offset from the backbone by a fixed gap. The lower panel shows a representative cross-section illustrating the DNA measurement window and the adjacent background regions used for quantitative analysis.


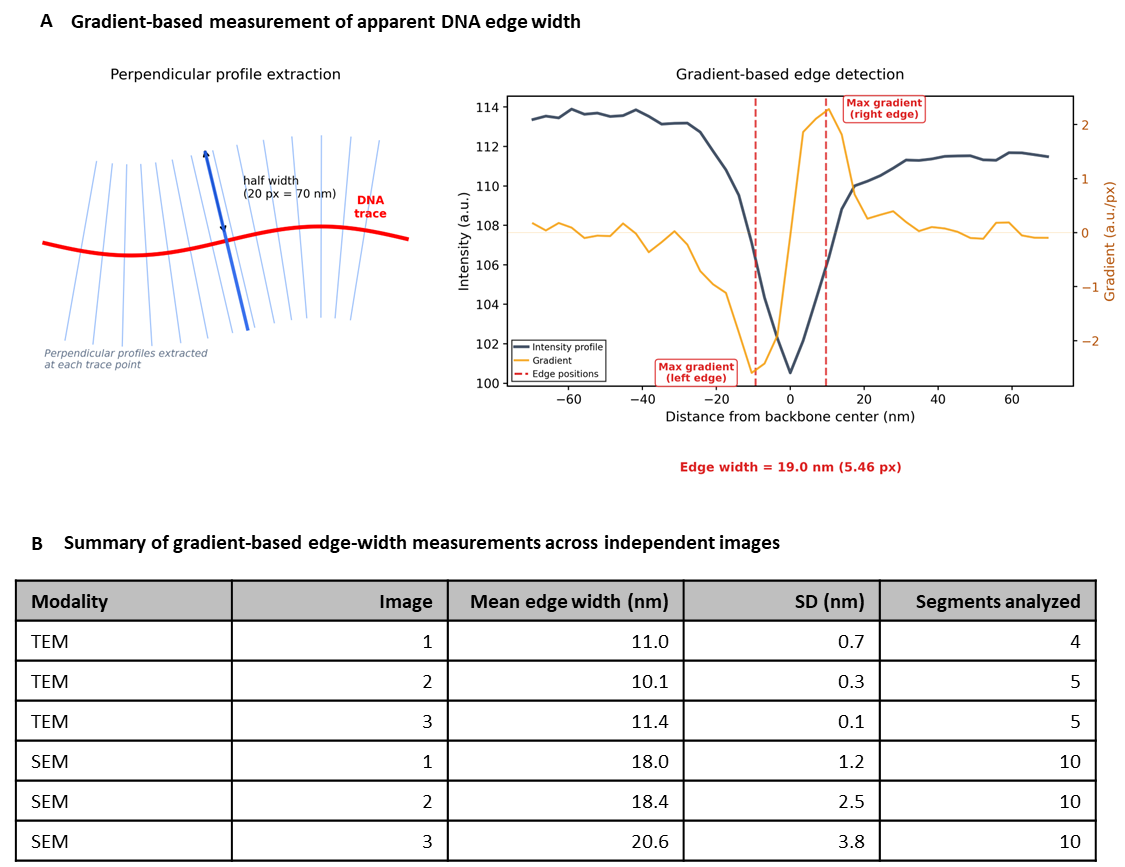


Figure S3. Gradient-based measurement of apparent DNA edge width from contour-aligned transverse profiles. (A) Perpendicular intensity profiles were extracted relative to the traced DNA backbone, and the apparent left and right edge positions were identified from the extrema of the first derivative of the transverse intensity profile. The distance between these two edge positions was used as a model-free measure of apparent DNA edge width. (B) Summary of gradient-based apparent edge-width measurements across independent TEM and SEM images. For each analyzed segment, 100 perpendicular profiles were extracted and averaged prior to gradient-based edge-width measurement. The table reports the mean edge width, standard deviation, and number of analyzed segments for each image.


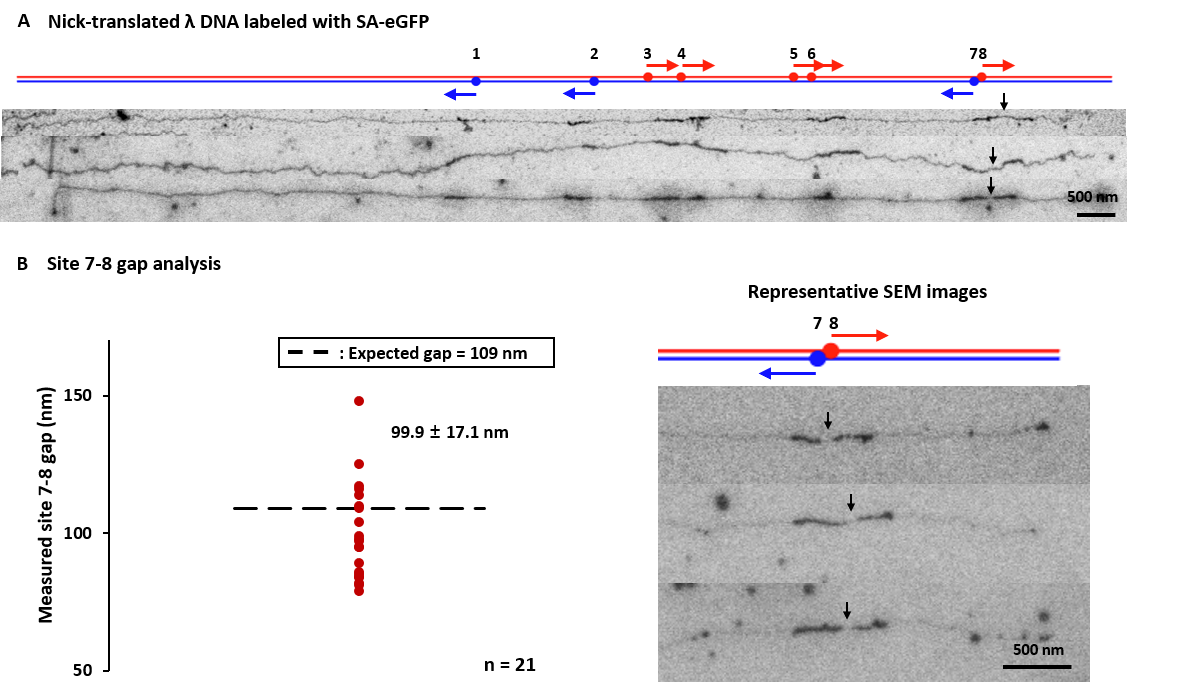


**Figure S4. Site 7–8 gap analysis of nick-translated λ DNA labeled with SA-FP. (A)** Representative SEM images of nick-translated λ DNA labeled with SA-eGFP. Arrows indicate the site 7–8 region. **(B)** Quantification of the measured gap between sites 7 and 8 from resolvable SA-FP-labeled molecules, including SA-RRvT- and SA-eGFP-labeled λ DNA. The dashed line indicates the expected gap of 109 nm. The measured gap was 99.9 ± 17.1 nm (mean ± SD, n = 21). The site 7–8 separation was resolved in 21 of 23 analyzed molecules (91%).

**
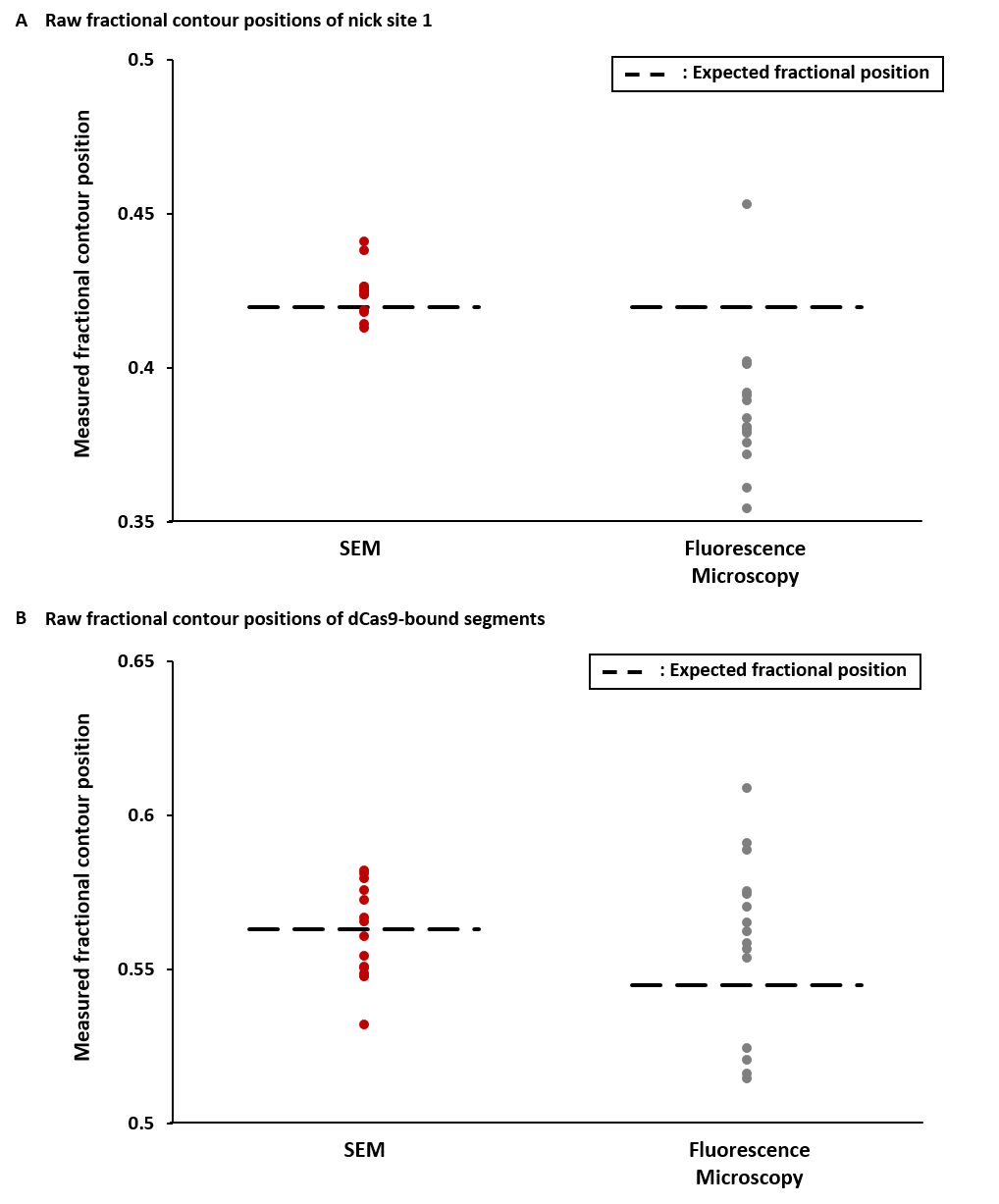
**

**Figure S5. Raw fractional contour positions of sequence-labeled sites. (A)** Raw fractional contour positions of nick site 1 in SA-FP-labeled λ DNA measured by SEM and fluorescence microscopy. **(B)** Raw fractional contour positions of dCas9-bound segments measured by SEM and fluorescence microscopy. Each dot represents an individual DNA molecule. Dashed lines indicate the expected fractional contour positions calculated from the reference DNA sequence. Fractional contour positions were calculated directly from raw contour-length measurements before conversion to bp-equivalent coordinates.

**
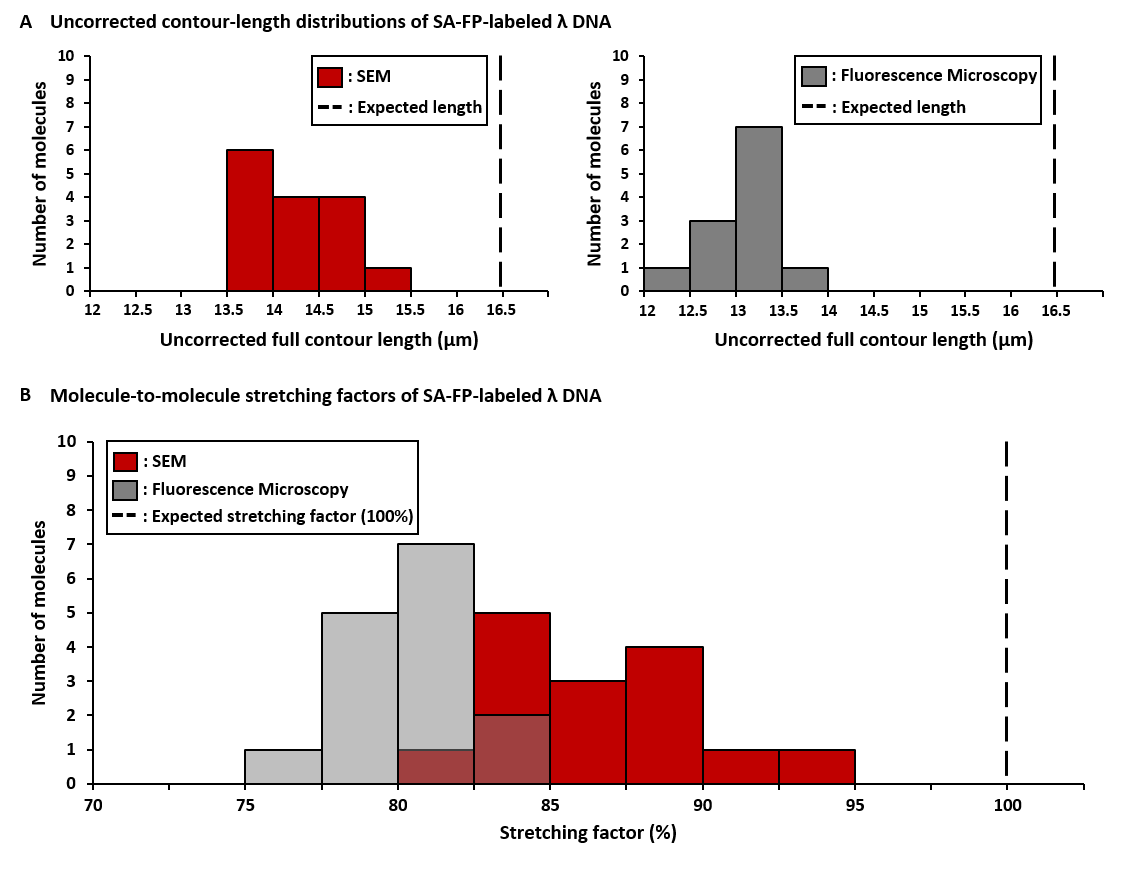
**

**Figure S6. Uncorrected contour-length distributions and stretching factors of SA-FP-labeled λ DNA. (A)** Distributions of uncorrected full contour lengths measured from SEM and fluorescence microscopy images of SA-FP-labeled λ DNA used for nick-site positional analysis. Dashed lines indicate the expected full contour length of λ DNA. **(B)** Molecule-to-molecule stretching factors calculated from the measured full contour lengths relative to the expected λ DNA contour length. The dashed line indicates the expected stretching factor of 100%.

**
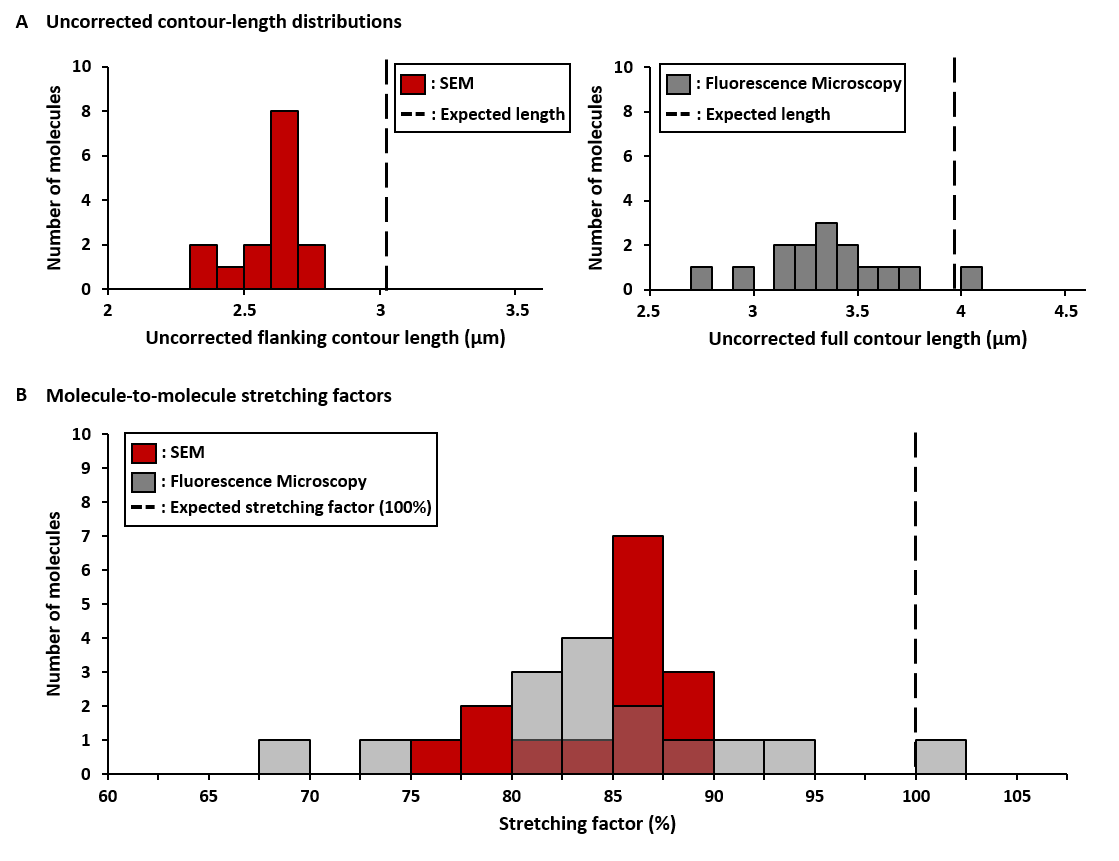
**

**Figure S7. Uncorrected contour-length distributions and stretching factors for dCas9-bound segment analysis. (A)** Uncorrected contour-length distributions of dCas9-labeled pWY82 DNA measured by SEM and fluorescence microscopy. SEM contour lengths represent the measurable left and right flanking arms after excluding the compacted dCas9-bound repeat segment, whereas fluorescence microscopy contour lengths represent full molecular contours. Dashed lines indicate the corresponding expected reference lengths. **(B)** Molecule-to-molecule stretching factors calculated from the measured contour lengths relative to the expected reference lengths. The dashed line indicates the expected stretching factor of 100%.


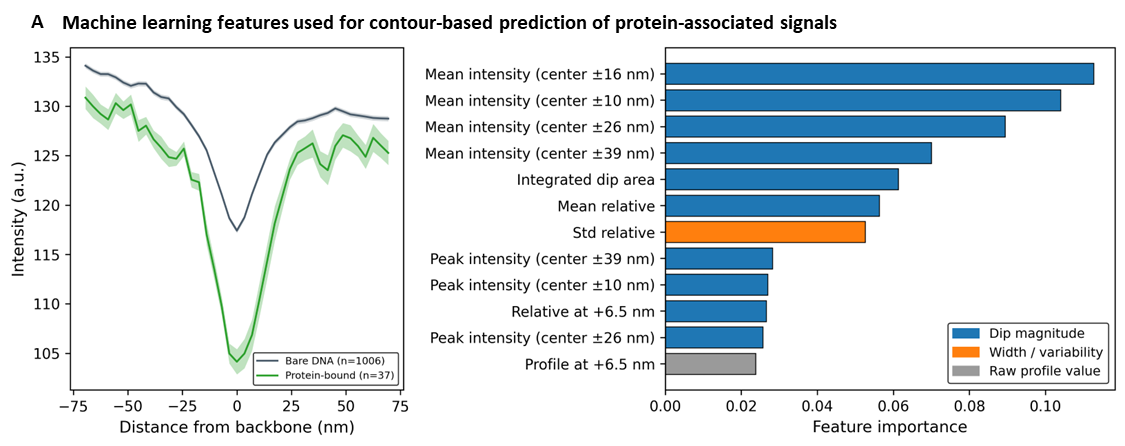


**Figure S8. Machine learning features used for contour-based prediction of protein-associated signals. (A)** Machine learning features used for contour-based prediction of protein-associated signals. Mean transverse intensity profiles of protein-free DNA and protein-associated signals used to illustrate the profile characteristics captured by the contour-based prediction framework. The plot on the left compares the average profiles, and the bar graph on the right summarizes the relative importance of representative features derived from profile intensity, dip magnitude, width, and variability. These features were used to distinguish protein-associated signals from protein-free DNA profiles.


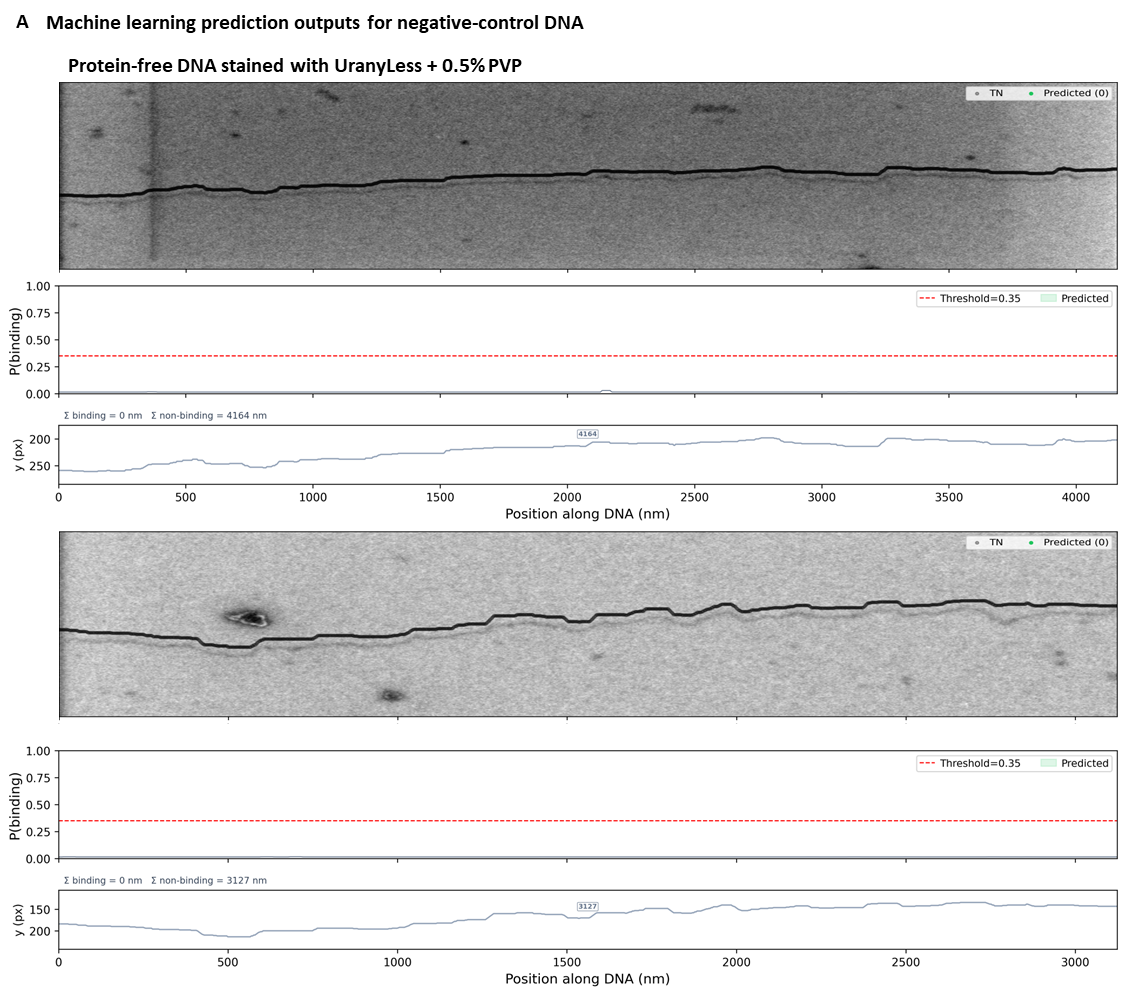


**Figure S9. Machine learning prediction outputs for negative-control DNA. (A)** Representative examples of protein-free DNA stained with UranyLess + 0.5% PVP and the corresponding machine learning prediction outputs. Traced DNA backbones are shown together with the predicted probability of protein-associated signal presence along the contour. In these negative-control molecules, the prediction scores remained below the decision threshold and no protein-associated regions were identified.


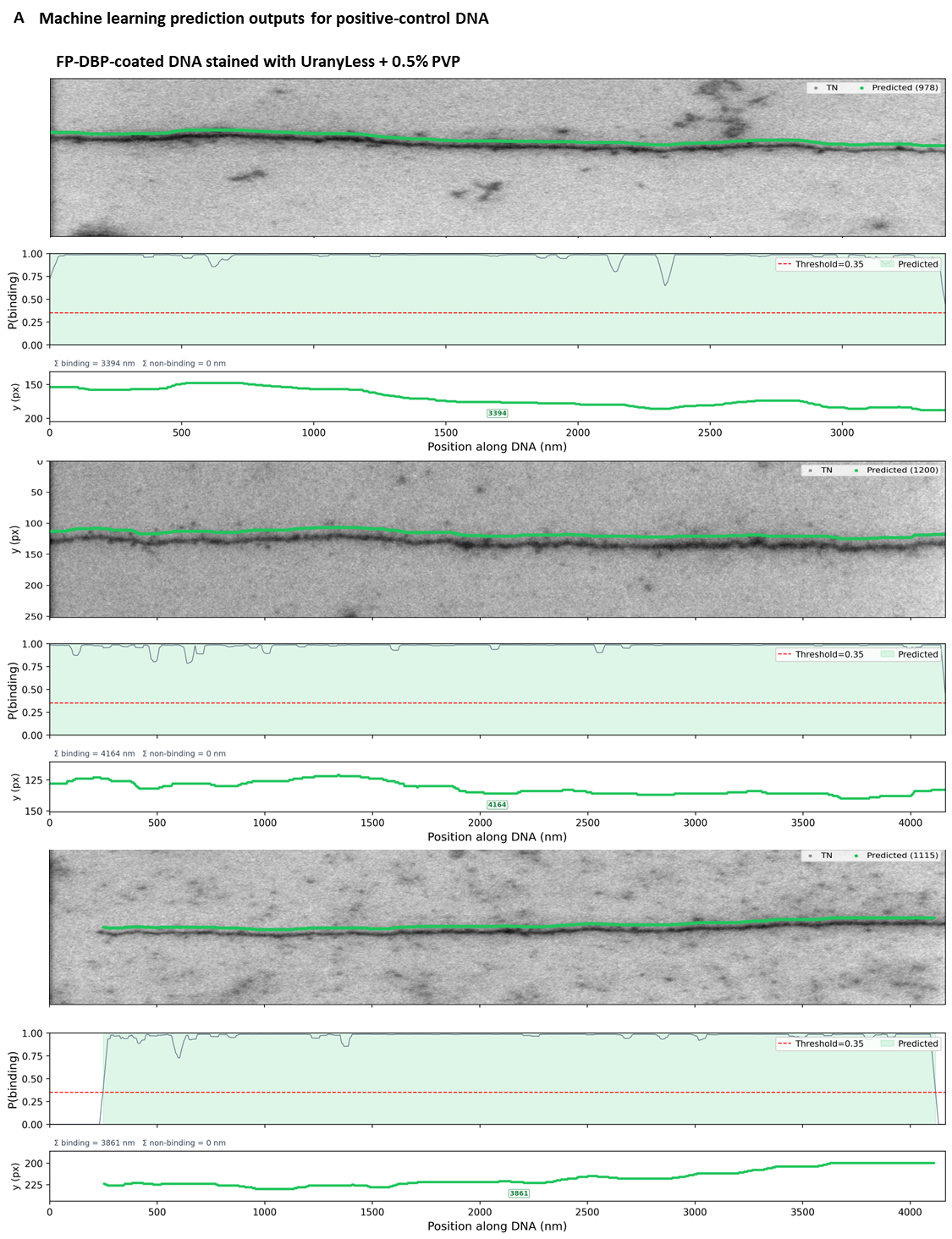


Figure S10. Machine learning prediction outputs for positive-control DNA. (A) Representative examples of FP-DBP-coated DNA stained with UranyLess + 0.5% PVP and the corresponding machine learning prediction outputs. Traced DNA backbones are shown together with the predicted probability of protein-associated signal presence along the contour. In these positive-control molecules, the prediction scores remained above the decision threshold over most of the traced contour, consistent with continuous protein-associated signals along the DNA.


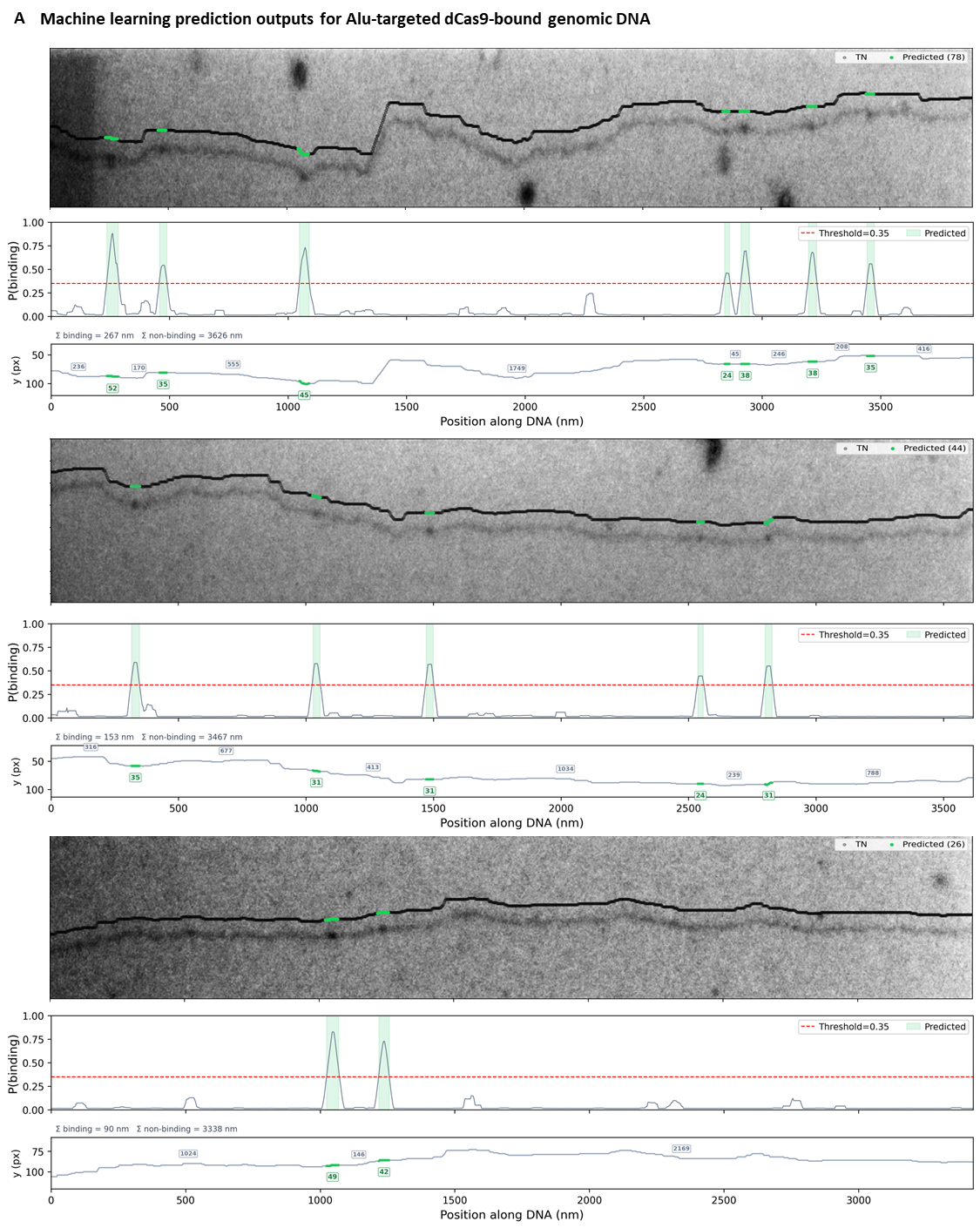


Figure S11. Machine learning prediction outputs for Alu-targeted dCas9-bound genomic DNA. (A) Representative examples of Alu-targeted dCas9-bound genomic DNA and the corresponding machine learning prediction outputs. Traced DNA backbones are shown together with the predicted probability of protein-associated signal presence along the contour. Green segments indicate positions predicted as protein-associated signal, and shaded regions in the probability plots indicate positions above the decision threshold. These examples illustrate detection of sparse localized dCas9-associated features along individual genomic DNA molecules.


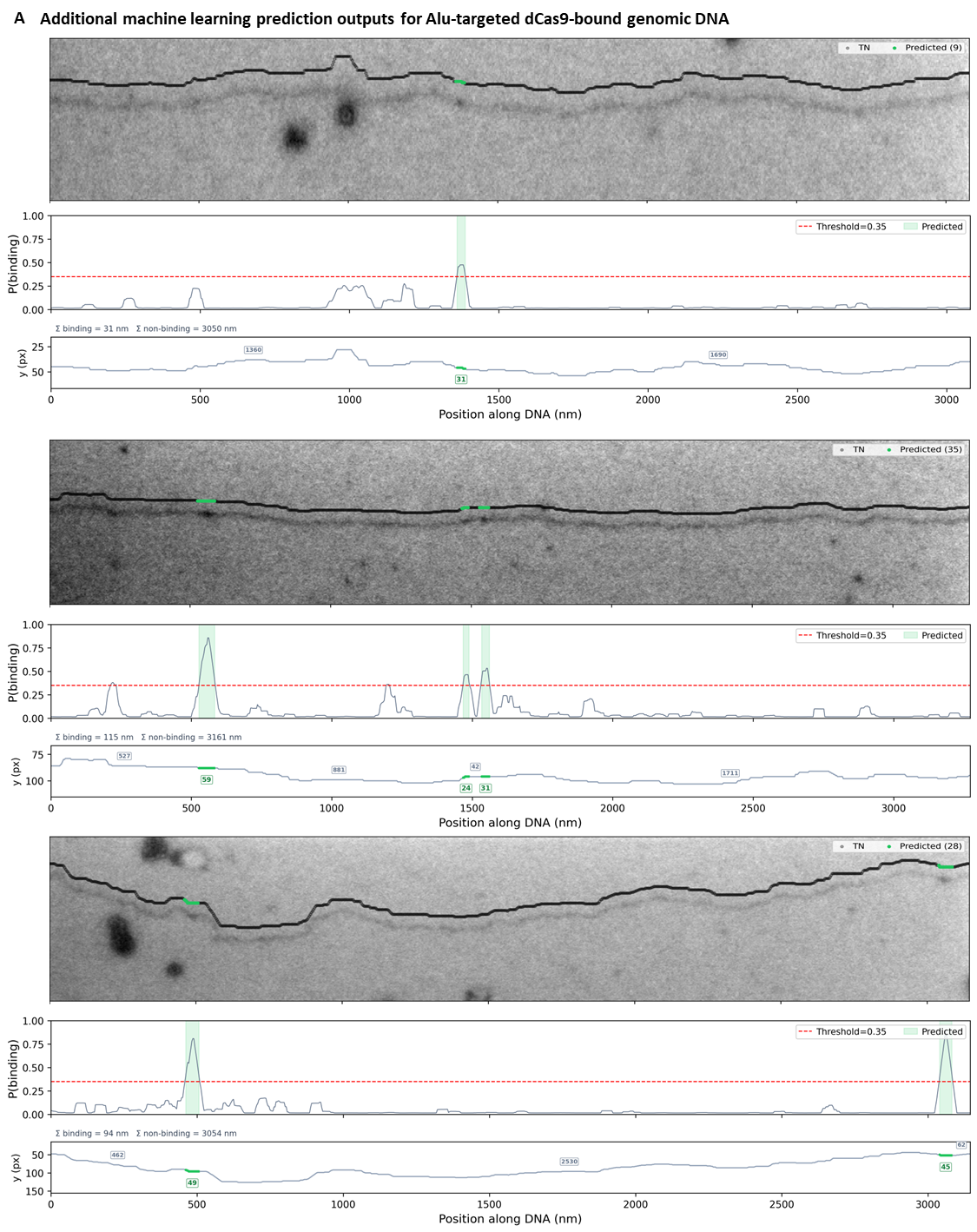


**Figure S12. Additional machine learning prediction outputs for Alu-targeted dCas9-bound genomic DNA. (A)** Additional representative examples of Alu-targeted dCas9-bound genomic DNA and the corresponding machine learning prediction outputs. Traced DNA backbones are shown together with the predicted probability of protein-associated signal presence along the contour. Green segments indicate positions predicted as protein-associated signal, and shaded regions in the probability plots indicate positions above the decision threshold. These additional examples further support detection of sparse localized dCas9-associated features along individual genomic DNA molecules.


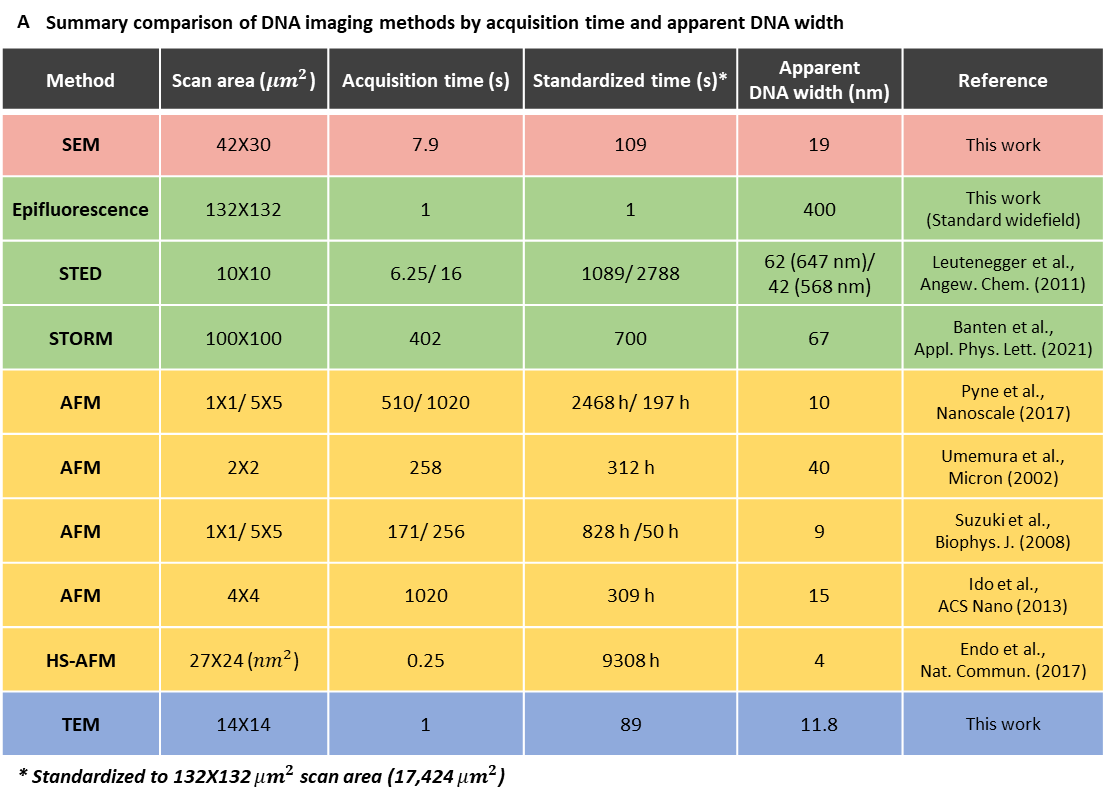


**Figure S13. Summary comparison of DNA imaging methods by acquisition time and apparent DNA width. (A)** Scan area, acquisition time, acquisition time standardized to the epifluorescence imaging area, and apparent DNA width are summarized for SEM, epifluorescence microscopy, STED, STORM, AFM, HS-AFM, and TEM using representative literature reports and data from this work. The epifluorescence imaging area (132 × 132 μm²) was used as the reference area for standardization, and the standardized acquisition time was calculated as the time required for each method to image the same area. Apparent DNA width refers to the experimentally observed signal width in each imaging modality.

**Supplementary Dataset. Raw quantitative data underlying Figures 1, 2, 3, and SI Figure 1.**

**Supplementary dataset is provided as an excel file.**

1. Kim, Y.T., Oh, H., Seo, M.J., Lee, D.H., Shin, J., Bong, S., Heo, S., Hapsari, N.D. and Jo, K. (2022) 21 Fluorescent Protein-Based DNA Staining Dyes. *Molecules*, **27**.

2. Shin, E., Kim, W., Lee, S., Bae, J., Kim, S., Ko, W., Seo, H.S., Lim, S., Lee, H.S. and Jo, K. (2019) Truncated TALE-FP as DNA Staining Dye in a High-salt Buffer. *Sci Rep-Uk*, **9**, 17197.

3. Jin, Y., Bae, J., Kim, T.Y., Hwang, H., Kim, T., Yu, M., Oh, H., Hashiya, K., Bando, T., Sugiyama, H. *et al.* (2022) Twelve Colors of Streptavidin-Fluorescent Proteins (SA-FPs): A Versatile Tool to Visualize Genetic Information in Single-Molecule DNA. *Anal Chem*, **94**, 16927-16935.

4. Kim, T., Kim, S., Noh, C., Hwang, H., Shin, J., Won, N., Lee, S., Kim, D., Jang, Y., Hong, S.J. *et al.* (2023) Counting DNA molecules on a microchannel surface for quantitative analysis. *Talanta*, **252**, 123826.

5. Kim, T. and Jo, K. (2023) Microfluidic Device to Maximize Capillary Force Driven Flows for Quantitative Single-Molecule DNA Analysis. *Biochip J.*, **17**, 384-392.

6. Zhou, S., Deng, W., Anantharaman, T.S., Lim, A., Dimalanta, E.T., Wang, J., Wu, T., Chunhong, T., Creighton, R., Kile, A. *et al.* (2002) A whole-genome shotgun optical map of Yersinia pestis strain KIM. *Appl Environ Microbiol*, **68**, 6321-6331.

7. Noh, C., Kang, Y.J., Heo, S., Kim, T., Kim, H., Chang, J., Sundharbaabu, P.R., Shim, S., Lim, K.I., Lee, J.H. *et al.* (2024) Scanning Electron Microscopy Imaging of Large DNA Molecules Using a Metal-Free Electro-Stain Composed of DNA-Binding Proteins and Synthetic Polymers. *Advanced Science*, **11**, 2309702.
